# Engineering Plant-Associated Soil Microbiomes for Sustainable and Climate-Resilient Agriculture: Mechanisms, Technologies, and Applications

**DOI:** 10.3390/microorganisms14081648

**Published:** 2026-07-28

**Authors:** Amankeldi K. Sadanov, Gul Baimakhanova, Baiken B. Baimakhanova, Saltanat Orazymbet, Irina Ratnikova, Irina Smirnova, Nurgul Mamytova, Raikhan Sydykbekova, Bekzhan D. Kossalbayev, Gulzat S. Aitkaliyeva, Ayaz M. Belkozhayev

**Affiliations:** 1 LLP “Research and Production Center for Microbiology and Virology”, Almaty 050010, Kazakhstan; a.sadanov1951@gmail.com (A.K.S.); bgulb@mail.ru (G.B.); bbbayken@mail.ru (B.B.B.); saltanatmikro@gmail.com (S.O.); iratnikova@list.ru (I.R.); iesmirnova@mail.ru (I.S.); 2 Department of Biotechnology, Faculty of Biology and Biotechnology, Al-Farabi Kazakh National University, Almaty 050040, Kazakhstan; mamytovanur@gmail.com (N.M.); raihan_07_77@mail.ru (R.S.); 3 Department of Chemical and Biochemical Engineering, Geology and Oil-Gas Business Institute Named After K. Turyssov, Satbayev University, Almaty 050043, Kazakhstan; kossalbayev.bekzhan@gmail.com; 4 Ecology Research Institute, Khoja Akhmet Yassawi International Kazakh Turkish University, Turkistan 161200, Kazakhstan; 5 M.A. Aitkhozhin Institute of Molecular Biology and Biochemistry, Almaty 050012, Kazakhstan

**Keywords:** plant-associated soil microbiome, rhizosphere microbiome, microbiome manipulation, microbiome engineering, plant–microbe interactions, synthetic microbial communities, sustainable agriculture

## Abstract

Soil microbiomes are essential for nutrient cycling, plant health, stress resilience, and sustainable agriculture. Recent advances in high-throughput sequencing, multi-omics technologies, systems biology, and artificial intelligence (AI) have transformed our understanding of plant–microbiome interactions and enabled the development of innovative microbiome engineering strategies. This review provides a comprehensive overview of the mechanisms governing plant-associated soil microbiome assembly, microbial community functions, plant–microbe communication, and microbiome-mediated stress resistance in agricultural ecosystems. Current approaches to plant-associated soil microbiome manipulation and engineering, including microbial inoculants, synthetic microbial communities (SynComs), microbiome transplantation, rhizosphere steering, and synthetic biology-based interventions, are critically examined. The review further discusses the growing role of metagenomics, metabolomics, metatranscriptomics, machine learning (ML), and precision agriculture technologies in improving microbiome characterization, prediction, and management. Particular attention is given to the application of microbiome-based solutions for sustainable crop production, nutrient management, biological control, climate-smart agriculture, and ecosystem restoration. Despite significant progress, challenges related to field-scale variability, colonization stability, biosafety, regulatory frameworks, and data integration continue to limit large-scale implementation. Future advances in precision microbiome engineering are expected to combine ecological principles, multi-omics technologies, AI, and synthetic biology to develop predictive and resilient microbiome-based solutions for sustainable and climate-resilient agriculture.

## 1. Introduction

Agricultural systems are facing unprecedented challenges associated with climate change, soil degradation, biodiversity loss, emerging plant diseases, and the growing demand for food production. Increasing temperatures, irregular precipitation patterns, salinization, nutrient depletion, and the intensive use of agrochemicals threaten both crop productivity and long-term ecosystem sustainability [1,2]. Traditional agricultural practices have largely focused on improving plant genetics, irrigation systems, and fertilizer management; however, growing evidence suggests that the soil microbiome represents an equally important but historically underutilized component of crop production systems [3,4]. The soil microbiome comprises diverse communities of bacteria, archaea, fungi, protists, and viruses that collectively regulate essential ecosystem processes, including nutrient cycling, organic matter decomposition, carbon sequestration, soil aggregation, pathogen suppression, and plant stress adaptation [5]. These microbial communities occupy a central position at the interface between plants and soil, where they influence plant growth through a wide range of mechanisms, including biological nitrogen fixation, phosphorus solubilization, phytohormone production, induction of systemic resistance, and modulation of plant immune responses [5,6]. Consequently, the soil microbiome is increasingly recognized as a key determinant of agricultural productivity, resilience, and sustainability [7]. Recent advances in high-throughput sequencing, metagenomics, metatranscriptomics, metabolomics, and systems biology have transformed our understanding of soil microbial ecology [8,9]. Rather than viewing microbial communities as passive inhabitants of soil environments, current research demonstrates that microbiomes are dynamic and highly responsive biological networks that continuously interact with plants, environmental conditions, and agricultural management practices [10]. These discoveries have shifted scientific attention from describing microbial diversity toward understanding microbial functions, ecological interactions, and their potential manipulation for agricultural benefit [11,12]. The concept of soil microbiome engineering has emerged from this paradigm shift. Microbiome engineering refers to the deliberate modification, management, or design of microbial communities to enhance desirable ecosystem services, including nutrient-use efficiency, disease suppression, crop productivity, and tolerance to abiotic stresses [13]. Approaches such as microbial inoculants, synthetic microbial consortia, microbiome transplantation, rhizosphere engineering, host-mediated microbiome selection, and synthetic biology-based interventions are increasingly being explored as sustainable alternatives or complements to conventional agricultural inputs [14]. At the same time, AI, ML, and multi-omics technologies are providing unprecedented opportunities to predict microbiome behavior and develop precision microbiome management strategies [15]. Despite remarkable progress, significant challenges remain. Field-scale variability, ecological instability of introduced microorganisms, limited understanding of microbial interactions, regulatory constraints, and difficulties in translating laboratory findings into practical agricultural applications continue to restrict large-scale implementation of microbiome-based technologies [16,17]. Addressing these challenges requires an integrated understanding of microbiome assembly, function, engineering strategies, and their application under real-world agricultural conditions [18,19,20]. Therefore, this review provides a comprehensive overview of recent advances in the study, manipulation, and engineering of plant-associated soil microbiomes for sustainable and climate-resilient agriculture. Emphasis is placed on rhizosphere- and root-associated microbial communities, plant–microbiome interactions, mechanisms of microbiome assembly and function, emerging intervention strategies, and the application of multi-omics and AI technologies. The review focuses specifically on microbial communities directly associated with plant performance and agricultural management rather than providing an exhaustive analysis of all components of the broader soil biota. Finally, current limitations and future research directions for precision microbiome manipulation and engineering are critically discussed.

## 2. Plant-Associated Soil Microbiomes and Plant Interactions

The soil microbiome should be viewed as a highly dynamic and spatially organized ecological system rather than a simple catalogue of microbial taxa [21]. Within the soil–plant interface, microorganisms continuously interact with soil nutrients, plant-derived metabolites, and competing microbial populations, forming a biologically active environment that changes across different compartments [22]. Along the transition from bulk soil to the rhizosphere and subsequently into internal root tissues, microbial diversity generally decreases, whereas the selective influence of the host plant becomes increasingly pronounced [23]. This organization is of major agricultural importance because microbiome composition strongly influences nutrient uptake efficiency, pathogen resistance, and tolerance to environmental stress, thereby affecting crop productivity and long-term agroecosystem stability [24,25]. In recent years, research has progressed beyond purely descriptive analyses of microbial communities toward mechanistic and experimentally driven approaches integrating multi-omics technologies [26,27]. These studies have provided deeper insight into how plants selectively recruit beneficial microorganisms, restructure microbial communities during development or stress, and under certain environmental conditions partially lose regulatory control over their associated microbiota.

**Figure 1:**
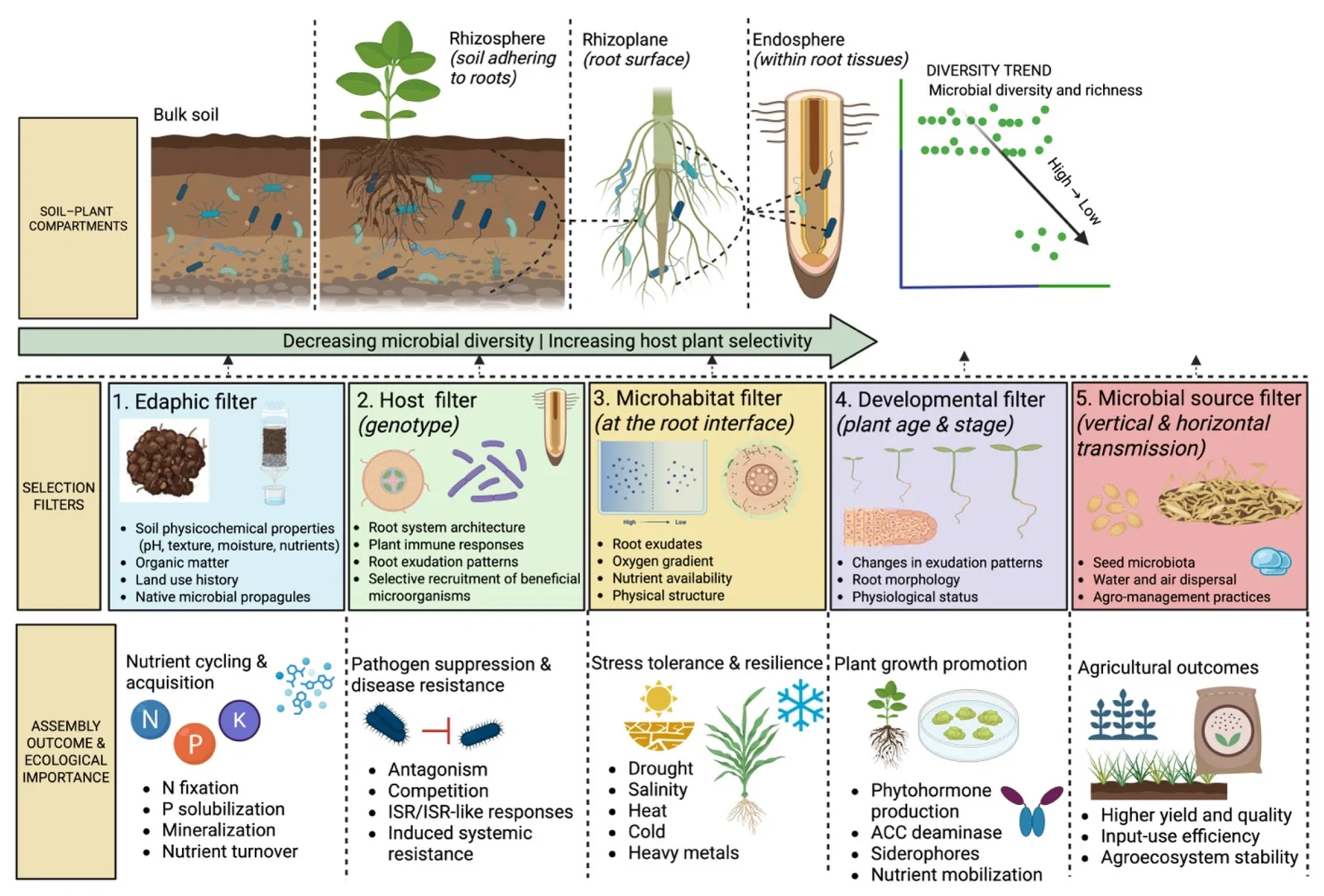
Soil–plant microbiome assembly across the soil–plant continuum. Arrows indicate microbial movement and host selectivity; green dots show declining diversity; the red T-bar indicates pathogen inhibition; and N, P, and K denote nitrogen, phosphorus, and potassium. Created in BioRender, https://BioRender.com/31bzk8r (access on 23 July 2026).

As summarized in Figure 1, plant-associated microbiome assembly proceeds along the bulk soil–rhizosphere–rhizoplane–endosphere continuum, where microbial diversity generally decreases and host selectivity increases. Edaphic, host-genetic, developmental, microhabitat, and microbial-source filters jointly determine community composition and functional outcomes related to nutrient acquisition, disease suppression, stress tolerance, and plant growth.

### 2.1. Structure and Diversity of the Soil Microbiome

Conceptually, soil microbiome organization can be viewed as the outcome of sequential ecological selection processes operating across spatial and temporal scales [28]. The initial level of selection is determined by the edaphic reservoir, where soil physicochemical properties, nutrient status, historical land use, and resident microbial propagules define the taxa potentially available for colonization [29,30]. A second level of filtering is imposed by the host plant, which selectively recruits microorganisms into distinct microhabitats, including the rhizosphere, rhizoplane, and endosphere [31]. A third dynamic component is developmental timing, since plant age and physiological status continuously reshape root exudation patterns and, consequently, microbial recruitment [32]. Understanding these layered interactions is scientifically important because microbial diversity at the root–soil interface directly influences the range of functional processes that can occur within agricultural ecosystems, including nutrient cycling, pathogen suppression, and stress resilience [33,34].

A clear example of compartment-specific microbiome assembly was provided by Brown et al. (2020), who investigated three *Medicago truncatula* genotypes cultivated in two contrasting native soils [35]. The authors found that bacterial diversity progressively decreased from the rhizosphere to internal root compartments. Soil origin primarily shaped rhizosphere communities, whereas host genotype exerted a stronger influence within root-associated niches. These findings demonstrate that microbiome structure is jointly determined by environmental and host-related factors, with their relative importance varying across the soil–plant continuum.

From a crop improvement perspective, Kavamura et al. (2020) further demonstrated that wheat breeding history can influence rhizosphere microbiome composition [36]. By comparing tall and semi-dwarf cultivars, the study showed that variation in root architecture was associated with distinct bacterial community profiles. Tall cultivars were enriched in Actinobacteria, Bacteroidetes, and Proteobacteria, whereas semi-dwarf cultivars exhibited higher abundances of Verrucomicrobia, Planctomycetes, and Acidobacteria. These results suggest that conventional breeding has indirectly shaped rhizosphere microbiomes through the selection of plant morphological and physiological traits.

Extending this concept to domestication processes, Spor et al. (2020) showed that the evolutionary history of wheat also contributes to microbiome assembly [37]. Analysis of 39 tetraploid wheat accessions representing different domestication stages revealed significant differences in bacterial, fungal, and nitrogen-cycling microbial communities. Modern cultivars and ancestral germplasm differed in their associations with nitrogen-related microbial guilds, indicating that domestication has unintentionally modified plant–microbe interactions. These findings highlight the potential value of wild relatives and traditional landraces as reservoirs of beneficial microbiome-associated traits for future breeding programs.

In addition to host genetics and domestication, temporal variation represents another critical determinant of microbiome organization. Xiong et al. (2021) demonstrated that plant developmental stage had a stronger influence on microbial diversity, taxonomic composition, and interkingdom interaction networks within plant-associated compartments than in surrounding soils [38]. Bacterial communities predominated during the early stages of maize development, whereas fungal taxa became increasingly important later in the growing season. These shifts were accompanied by corresponding changes in predicted ecological functions and metabolic potential. Collectively, the results indicate that plant microbiomes are highly dynamic systems that continuously reorganize throughout host development, emphasizing the importance of temporal resolution in microbiome studies.

More recently, the role of microbial inheritance during microbiome establishment was highlighted by Garrido-Sanz et al. (2025) [39]. Using a sequential wheat rhizobiome propagation model in sterilized soil systems, the authors demonstrated that stable rhizosphere communities emerged through the coalescence of soil-derived and seed-associated microorganisms. Furthermore, seed-borne bacterial taxa were linked to saccharide metabolism pathways that facilitated early rhizosphere colonization and community establishment. These findings challenge the traditional soil-centered view of microbiome assembly and highlight the important role of vertically transmitted seed microbiota in shaping belowground microbial communities during the initial stages of plant development. The experimental designs, principal findings, and contributions of these representative studies are comparatively summarized in Table 1.

Taken together, these studies support a hierarchical but non-deterministic model of microbiome assembly in which soil origin, host genotype, domestication history, developmental stage, and microbial inheritance interact rather than operate independently. Soil properties appear to exert the strongest influence on the rhizosphere community, whereas host filtering becomes increasingly important in the rhizoplane and endosphere. However, the relative contribution of each factor remains difficult to compare across studies because of differences in crop species, soil types, sampling compartments, sequencing approaches, and experimental scales. In addition, several studies relied primarily on amplicon-based community profiling or functional prediction, which identifies associations but does not necessarily demonstrate causal microbial functions. Experiments conducted in sterilized soils or controlled microcosms provide stronger mechanistic resolution but may underestimate competition, dispersal limitation, and environmental heterogeneity under field conditions. Therefore, an unresolved question is whether general assembly rules can be transferred across crops and soils or whether microbiome organization remains predominantly site- and host-specific [35,36,37,38,39].

### 2.2. Functional Roles in Agroecosystems

The ecological importance of the soil microbiome in agriculture lies not only in who is present, but in what the community collectively does. In crop systems, the most consequential functions are nutrient mobilization and transformation, disease suppression, resilience to management disturbance, and yield support under nutrient or pathogen pressure [40,41]. Scientifically, this subsection is central because it shifts the discussion from diversity as pattern to microbiome activity as process [42].

A notable example of function-oriented microbiome research was provided by Wang et al. (2024) in soybean grown under long-term nutrient imbalance conditions [43]. Using quantitative microbiome profiling across eight developmental stages, the study demonstrated that nutrient deficiency altered not only microbial community composition but also microbial abundance and succession patterns. Under nitrogen omission, a distinct low-nitrogen-enriched ecological cluster became dominant, and a synthetic community derived from this cluster promoted plant growth, corresponding to the observed maintenance of soybean productivity in the absence of nitrogen fertilization. These findings directly linked microbiome dynamics to agronomic performance rather than merely describing taxonomic variation.

Another important contribution addressed the carbon-cycle dimension of rhizosphere functioning. In a long-term maize fertilization experiment established in 1984, metagenomic analyses identified eight carbon-fixation pathways and revealed that the reductive tricarboxylic acid (rTCA) cycle dominated carbon fixation within the maize rhizosphere. Nitrogen fertilization increased the abundance of key rTCA-related genes and stimulated acetate-dependent methanogenesis, whereas straw-return practices enhanced genes associated with carbon degradation. Soil organic carbon and nitrate availability emerged as major drivers of these functional patterns, highlighting the role of rhizosphere microorganisms in regulating carbon transformation and methane-related processes [44].

Beyond nutrient cycling, management practices can also influence microbiome function throughout the soil profile. Using depth-stratified analyses combining 16S rRNA gene and internal transcribed spacer (ITS) sequencing, machine learning approaches, and microbial assembly models, it was shown that fertilizer source represented the dominant management factor, particularly for fungal communities. In contrast, tillage primarily affected the upper soil layer by increasing microbial dispersal and stochastic assembly processes. These results demonstrate that microbiome-mediated functions such as nutrient cycling, pathogen buffering, and community connectivity vary substantially with soil depth [45].

Disease suppression represents another critical ecosystem service provided by soil microbial communities. Integrating field surveys, microbiome transfer experiments, metagenomics, and metabolomics, recent work demonstrated that soil acidification significantly weakened the capacity of indigenous microbiomes to suppress *Fusarium* infection. Acidified soils exhibited reduced expression of sulfur-metabolism-related functions, and microbiomes originating from these soils displayed lower antagonistic activity against pathogens both in vitro and during plant inoculation experiments. These findings suggest that disease suppressiveness is not an inherent property of all soils but rather a functionally contingent characteristic influenced by environmental conditions such as pH [46].

The functional scope of microbiome research has also expanded to include plant defense and pest regulation. Analysis of 136 organically managed farm soils revealed that practices such as no-tillage, targeted irrigation, and grass cover cropping were associated with microbial shifts linked to enhanced jasmonic acid and salicylic acid signaling as well as reduced aphid abundance. In contrast, compost application produced more variable outcomes, indicating that not all biologically based management strategies enhance microbiome-mediated defense mechanisms to the same extent. These findings provide experimental evidence connecting soil microbial communities with aboveground plant protection processes [47].

More recently, attention has shifted toward multi-kingdom interactions underlying microbiome function. Combining bacterial and fungal amplicon sequencing with untargeted metabolomics in ginger cultivars revealed that high-yielding plants supported more complex microbial interaction networks and were enriched in keystone taxa such as *Talaromyces* and *Devosia*. Several host-derived metabolites, including Niazimin A and 1-oleoyl-lysophosphatidic acid, showed positive associations with nitrogen-fixing and plant growth-promoting microorganisms. This study highlights that microbiome function emerges from coordinated interactions among plants, metabolites, and microbial networks rather than from the presence of individual beneficial taxa alone [48].

An even broader ecological perspective was recently introduced through the study of soil food-web interactions. Disease suppression following biofertilizer application was associated with increased abundance of predatory protists, particularly *Cercomonas*, together with enrichment of genes involved in secondary metabolite biosynthesis, including polyketide synthases. These results indicate that microbiome-mediated suppressiveness can emerge not only from direct microbial antagonism but also from trophic interactions and network-level ecological reorganization within the soil ecosystem [49]. Plant-associated soil microbiomes enhance stress tolerance under drought, salinity, heat, and nutrient limitation by improving nutrient uptake, root development, hormone balance, osmotic regulation, antioxidant defense, and soil water retention. However, these effects depend on plant genotype, soil properties, microbial composition, and stress severity [2,33,34]. Collectively, these studies indicate that microbiome functions in agroecosystems emerge through interacting mechanisms involving resource availability and nutrient transformation, physicochemical filtering by soil conditions, plant stress regulation, and network-level interactions among bacteria, fungi, plants, and microbial predators. The strongest causal evidence was obtained in studies combining field observations with microbiome transfer, plant bioassays, or SynCom validation, because these approaches tested whether an identified microbial community could reproduce the expected phenotype [43,46]. In contrast, co-occurrence networks, structural equation models, and correlations between microbial taxa and plant metabolites remain primarily hypothesis-generating and cannot independently distinguish whether microbial changes are the cause or consequence of improved plant performance [45,47,48]. The variable response to compost application and the pH dependence of disease suppression further demonstrate that a microbial practice that is beneficial in one environment may be neutral or ineffective in another. Thus, no universal taxonomic signature of a “beneficial microbiome” has yet emerged; functional outcomes depend on environmental context, community interactions, and experimental validation [43,44,45,46,47,48,49]. The experimental designs, principal mechanistic findings, and agronomic significance of these representative studies are comparatively summarized in Table 2.

**Figure 2:**
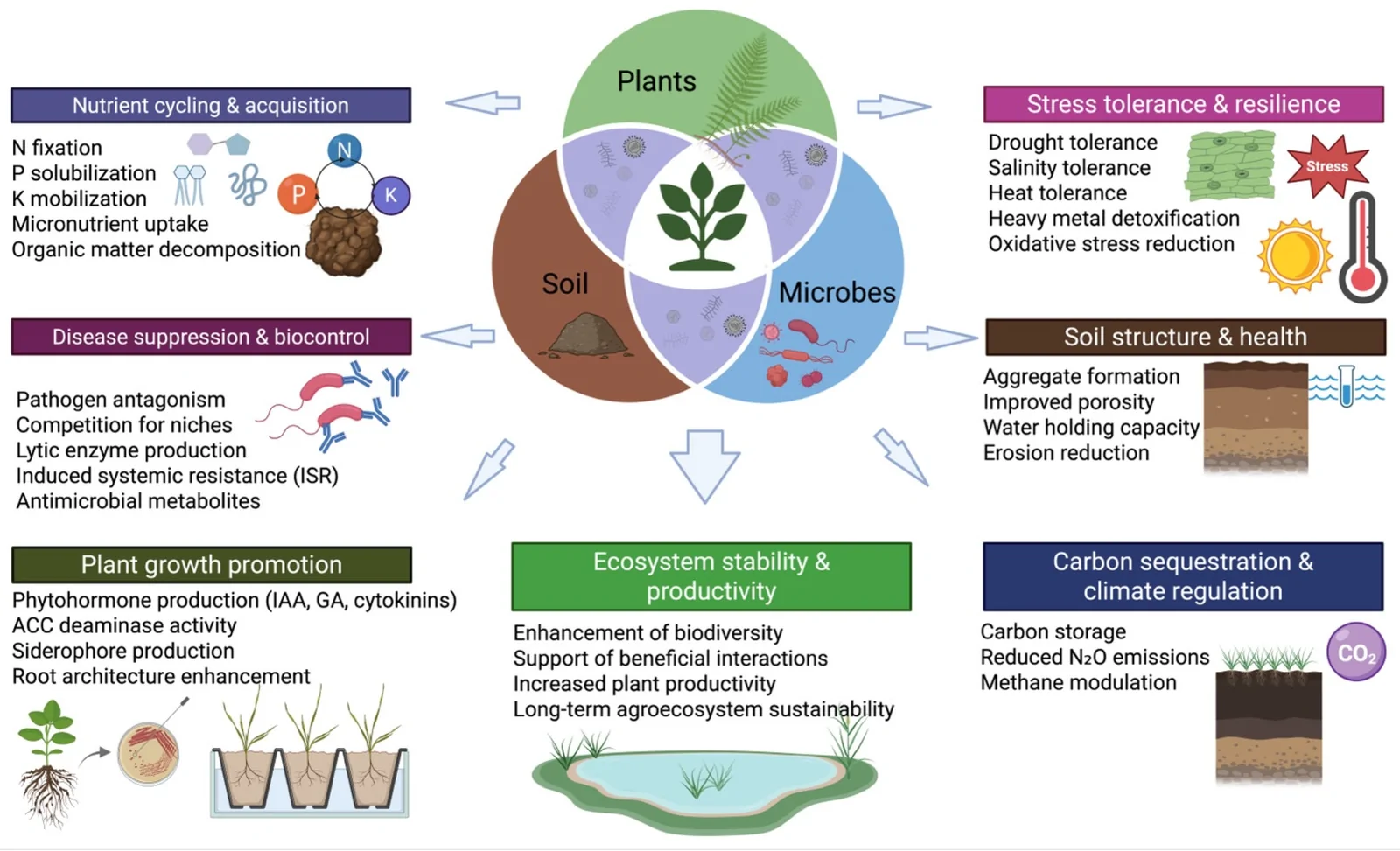
Functional roles of the soil microbiome in agroecosystems. Soil microorganisms contribute to nutrient cycling and acquisition, disease suppression, plant growth promotion, stress tolerance, soil structure maintenance, carbon sequestration, and ecosystem stability. Through interactions among plants, soil, and microbial communities, these functions collectively enhance plant productivity, resilience, and long-term agroecosystem sustainability. Outward arrows indicate that plant–soil–microbe interactions drive the major microbiome-mediated functions shown around the central diagram. Created in BioRender, https://BioRender.com/erkj4t4 (access on 23 July 2026).

The principal functional pathways through which plant-associated soil microbiomes influence agroecosystem performance are summarized in Figure 2. These functions include nutrient cycling and acquisition, pathogen suppression, plant growth promotion, stress tolerance, maintenance of soil structure, carbon sequestration, and ecosystem stability. Together, these processes illustrate how microbial activity is translated into crop productivity, resilience, and long-term sustainability.

### 2.3. Mechanisms of Plant–Microbiome Communication

Plant–microbiome communication is fundamentally chemical. Plants release exudates, volatiles, and defense-associated metabolites; microbes perceive these signals, move toward them, metabolize them, or sometimes subvert them [50,51]. The scientific importance of this topic is that communication mechanisms explain specificity: why one plant recruits one microbial consortium and excludes another, and why the same microbe can be beneficial in one metabolic context yet costly in another [52].

Microbe–microbe communication in the rhizosphere occurs through quorum sensing, volatile and secondary metabolites, and metabolic cross-feeding. These interactions regulate colonization, biofilm formation, nutrient exchange, competition, and pathogen suppression, allowing plant-associated functions to emerge at the community level rather than from individual strains alone [22].

A clear example of chemically mediated microbiome assembly was provided by Yu et al. (2021) in maize [53]. By integrating root transcriptomics, rhizosphere profiling, plant genetics, and bacterial assays, the study demonstrated that root-derived flavones function as active recruitment signals rather than passive exudates. Flavones preferentially enriched members of the Oxalobacteraceae under nitrogen-limited conditions, enhanced nitrogen acquisition, and interacted with the lateral-root regulator LRT1. These findings revealed a reciprocal relationship in which root architecture influences exudation patterns, exudates recruit specific microbial groups, and the recruited microbiota subsequently affect plant performance under nutrient stress.

The role of host signaling in microbiome assembly was further explored by Tao et al. (2024) [54]. Using *Lotus japonicus* mutants impaired at different stages of Nod factor perception, together with gnotobiotic systems and metabolomic analyses, the study showed that symbiotic signaling alters root exudate composition and consequently reshapes root-associated bacterial communities. Importantly, microbiome assembly differed among nitrogen-starved, symbiotic, and inorganic nitrogen-supplemented plants, indicating that host nutritional status strongly influences microbial recruitment. These findings demonstrate that rhizobial signaling extends beyond the rhizobium–legume interaction and contributes to broader microbiome restructuring through host-mediated chemical changes.

Communication processes are not restricted to plant–microbe interactions but may also occur between neighboring plants. Investigation of a potato-onion–tomato system revealed that volatile organosulfur compounds, particularly dipropyl disulfide, modified tomato root exudation patterns and increased the recruitment of beneficial *Pseudomonas* and *Bacillus* species. Through twin-chamber experiments, rhizosphere transplantation, metabolomics, and biofilm assays, it was demonstrated that volatile compounds acted indirectly by reprogramming host exudation rather than directly stimulating microbial growth. This study expanded the concept of microbiome communication to include airborne inter-plant signals that subsequently influence belowground microbial assembly [55].

In a tomato split-root system challenged with *Fusarium oxysporum* f. sp. *lycopersici*, pathogen-derived fusaric acid altered root exudation patterns and affected the recruitment of the disease-suppressive bacterium *Sphingomonas* sp. Sm12 [56]. Resistant cultivars recruited more suppressive microbial communities in response to fusaric acid, whereas susceptible cultivars exhibited reduced suppressiveness. These observations highlight that microbiome communication is simultaneously shaped by host genotype and pathogen activity, making the rhizosphere a dynamic arena of both cooperation and conflict.

Analysis of maize-associated bacteria identified the lactonase BxdA as a key determinant enabling microorganisms to metabolize benzoxazinoids, particularly MBOA, and utilize host-specialized metabolites as carbon sources. Bacteria capable of forming AMPO accumulated preferentially in the rhizosphere of benzoxazinoid-producing maize, whereas deletion of the *bxdA* gene abolished both MBOA utilization and AMPO production [57]. These findings demonstrate that microbial colonization depends not only on plant-derived signals but also on the metabolic capacity of microorganisms to exploit those signals. Recently, Gu et al. (2026) demonstrated that communication signals can be exploited by rhizosphere bacteria for their own ecological advantage [58]. A *Pseudomonas* strain isolated from the rhizosphere was able to degrade simple coumarins under iron-deficient conditions through redundant *xenA*-type determinants. Although this enhanced bacterial colonization, it simultaneously reduced plant iron acquisition and overall plant fitness. This study highlights an important limitation in microbiome engineering strategies: plant-derived metabolites do not inevitably promote beneficial microbial recruitment. Instead, microbial catabolism can transform host signaling molecules into mechanisms that favor microbial persistence at the expense of plant performance.

These findings collectively show that root exudates should not be viewed as universally beneficial recruitment signals, but rather as context-dependent ecological filters. In several systems, plant-derived flavones, volatiles, or stress-associated metabolites promoted the recruitment of microorganisms that improved nutrient acquisition or pathogen suppression [53,54,55,56]. In contrast, microbial degradation of coumarins under iron deficiency enhanced bacterial colonization while reducing plant iron acquisition and fitness [58]. This apparent contradiction indicates that the outcome of chemical communication depends on host genotype, nutrient status, microbial catabolic capacity, and competition among resident microorganisms. Methodologically, much of the current evidence originates from gnotobiotic systems, split-root experiments, mutant plants, or simplified microbial communities. Although these approaches are valuable for identifying causal mechanisms, it remains unclear whether individual chemical signals retain the same importance in heterogeneous field soils containing highly diverse microbial communities. Future studies should therefore combine targeted manipulation of root exudates with field-scale microbial tracking and functional validation.

### 2.4. Microbiome-Mediated Plant Stress Resistance

Stress resistance is the most agronomically consequential expression of plant–microbiome interaction because it translates microbial ecology into survival, reproduction, and yield under climate instability [59,60]. The underlying concept is that plants do not resist drought, salinity, or disease solely through intrinsic physiological mechanisms; they frequently do so by recruiting, maintaining, or even remembering beneficial microbial partners that enhance defense responses, improve osmotic regulation, modulate hormone signaling, and reshape the rhizosphere into a more protective environment [61]. Recent advances have significantly improved our mechanistic understanding of these processes by identifying specific metabolites, microbial genes, host traits, and microbiome legacy effects involved in stress adaptation [62,63].

A particularly elegant example of metabolite-mediated drought protection was reported by He et al. (2022) [64]. Through metabolite-dependent microbiome profiling, chemotaxis assays, transcriptomics, and dehydration experiments in *Arabidopsis*, the study demonstrated that flavonoids selectively attracted *Aeromonas* sp. H1. Colonization by this bacterium enhanced drought tolerance, increased survival, and improved photosynthetic performance under water deficit. Mechanistically, the protective effect was associated with enhanced guard-cell reactive oxygen species (ROS) accumulation, stomatal closure, and activation of jasmonic acid-related signaling pathways.

A complementary model of stress-responsive microbiome recruitment was later demonstrated under saline conditions. Integrated analyses combining amplicon sequencing, metagenomics, metatranscriptomics, bacterial isolation, chemotaxis assays, and mutant characterization revealed that salt stress stimulated the secretion of purines, particularly xanthine, by wild soybean roots. This metabolite promoted the enrichment of root-associated *Pseudomonas* populations, and two isolates significantly improved plant growth under saline conditions. Furthermore, chemotaxis-related genes, especially *cheW*, were essential for bacterial responses to xanthine [65]. The study established a direct mechanistic link between host metabolite secretion, microbial recruitment, and enhanced salinity tolerance.

Host developmental characteristics also influence the extent to which plants benefit from microbial protection. Using *Arabidopsis* mutants differing in root hair density, combined with multi-omics analyses and large-scale microbial isolation, it was shown that root hair developmental regulators strongly influence drought-induced microbiome assembly. Plants with increased root hair formation exhibited enhanced microbiome-mediated drought protection, whereas root hair-deficient mutants lost much of this benefit. Several *Rhizobium* strains displayed protective effects, and deletion of the *ocd* gene reduced their stress-alleviating capacity [66]. These findings indicate that microbiome-mediated drought tolerance is shaped not only by microbial traits but also by host developmental genetics.

The importance of microbiome legacy effects was highlighted by Vismans et al. (2022) [67]. The authors demonstrated that foliar infection by *Hyaloperonospora arabidopsidis* altered root exudation patterns and reshaped root-associated bacterial communities through coumarin-dependent mechanisms [67]. This process generated a soil-borne microbial legacy that enhanced salicylic acid-dependent defense responses in subsequent plants. Importantly, coumarin-deficient mutants failed to establish this protective legacy, demonstrating that microbiome-mediated defense can persist beyond the initial stress event and influence future plant generations.

Recent studies have also begun to differentiate the functional roles of microorganisms within complex stress-associated communities. Using a 13-week poplar mesocosm experiment subjected to drought, salinity, and pathogen stress, integrated analyses combining amplicon sequencing, metagenomics, network analysis, culturomics, and synthetic community experiments distinguished a stability-promoting core microbiota from stress-specific microbial assemblages. SynComs containing stress-responsive taxa improved plant tolerance, whereas core microorganisms primarily contributed to network robustness and community stability. These findings suggest that resilient microbiomes are composed of both stable and environmentally responsive microbial fractions [68]. Ginnan et al. (2025) demonstrated that stress resistance can depend strongly on microbiome history [69]. Across a precipitation gradient and a long-term drought experiment, microbial communities originating from low-precipitation environments mitigated drought-related physiological stress in a native grass species but not in maize. Transcriptomic analyses indicated that these legacy microbiota altered host pathways associated with transpiration and water-use efficiency. The results suggest that microbiome-mediated drought resilience can be historically contingent and may differ substantially between wild and domesticated plant species, potentially reflecting changes that occurred during crop domestication. Across these studies, microbiome-mediated stress resistance converges on several common mechanisms, including metabolite-guided microbial recruitment, modification of hormone and reactive oxygen species signaling, improved osmotic regulation, and stabilization of microbial interaction networks. Nevertheless, the protective effects were strongly dependent on host genotype, developmental traits, microbial history, and stress type. For example, drought-adapted legacy microbiomes alleviated stress in a native grass but not in maize, while root hair development determined the extent of microbiome-mediated protection in *Arabidopsis* [66,69]. These findings challenge the assumption that stress-associated microbiomes can be transferred uniformly among plant species. Furthermore, many studies were conducted under controlled conditions and evaluated short-term physiological traits rather than multi-season yield stability. Consequently, the persistence of these effects across soil types, crop genotypes, and combined field stresses remains insufficiently established [64,65,66,67,68,69].

## 3. Strategies for Soil Microbiome Manipulation and Engineering

In this review, microbiome manipulation and microbiome engineering are treated as related but distinct concepts. Microbiome manipulation refers to interventions that alter the composition or activity of an existing microbial community without deliberately redesigning its genetic or functional organization. Such approaches include microbial inoculation, microbiome transplantation, soil amendment, host-mediated recruitment, and modification of rhizosphere conditions. In contrast, microbiome engineering involves the rational design, reassembly, or genetic modification of microorganisms or microbial communities to achieve predefined functions. This category includes function-oriented SynCom design, synthetic biology, genome editing, and the construction of engineered microbial strains. Although the two concepts partially overlap, distinguishing them is important because they differ in their degree of control, predictability, ecological complexity, and regulatory requirements.

### 3.1. Microbial Inoculation and Synthetic Community-Based Approaches

Conventional inoculation is primarily a microbiome manipulation strategy because it introduces selected microorganisms into an existing community without fully redesigning the community. By contrast, function-oriented SynComs may represent an intermediate approach between manipulation and engineering, depending on whether strains are simply combined or rationally assembled according to predefined ecological functions. Microbial inoculants represent the most direct approach to soil microbiome engineering, involving the introduction of beneficial microorganisms or simple microbial consortia to enhance nutrient mobilization, suppress pathogens, or improve tolerance to abiotic stress [70]. In contrast, SynComs are intentionally designed microbial assemblages in which functional complementarity and ecological interactions among strains are as important as the characteristics of individual members. This distinction is particularly important because inconsistent field performance of microbial inoculants often results from ecological limitations, including poor establishment, insufficient functional redundancy, and weak resistance to competition from native microbiota [71,72].

#### 3.1.1. Plant-Growth-Promoting Bacteria and Their Functional Mechanisms

Plant-growth-promoting bacteria (PGPB), also commonly referred to as plant-growth-promoting rhizobacteria (PGPR), represent one of the most extensively studied groups of microbial inoculants for sustainable agriculture. Important representatives include species of *Bacillus*, *Pseudomonas*, *Paenibacillus*, *Azospirillum*, *Rhizobium*, and *Streptomyces*. However, plant-beneficial properties are generally strain-specific and should not be attributed uniformly to all members of a particular genus [73,74].

PGPB directly promote plant growth through biological nitrogen fixation, phosphate solubilization, siderophore-mediated iron acquisition, and the production of phytohormones such as indole-3-acetic acid. Bacteria possessing 1-aminocyclopropane-1-carboxylate (ACC) deaminase can also reduce stress-induced ethylene accumulation, thereby supporting root development and plant performance under drought, salinity, nutrient deficiency, and other adverse conditions [75,76].

Indirect mechanisms include pathogen suppression through the production of antibiotics, hydrolytic enzymes, volatile organic compounds, and iron-chelating siderophores. Beneficial bacteria may also compete with pathogens for nutrients and colonization sites, form biofilms on root surfaces, and induce systemic resistance in the host plant. These mechanisms allow PGPB to function simultaneously as biofertilizers, stress-protective agents, and biological control organisms. For example, characterization of the antibiotic-producing capabilities of *Lysobacter capsici* AZ78 demonstrated that bacterial biocontrol agents can be evaluated through their antimicrobial metabolite profiles rather than solely through plant growth responses [77,78,79].

Several studies discussed in this review illustrate these bacterial functions. Plant-mediated changes in root exudation increased the recruitment and biofilm formation of beneficial *Pseudomonas* and *Bacillus* species [55]. Salt-induced recruitment of *Pseudomonas* improved soybean performance under salinity, whereas selected *Rhizobium* strains enhanced microbiome-mediated drought protection [65,66]. These findings demonstrate that bacterial performance depends not only on the presence of beneficial traits but also on effective root recruitment, colonization, and compatibility with the host plant.

Nevertheless, the presence of a recognized plant-beneficial genus does not guarantee a positive agronomic outcome. Bacterial performance depends on strain identity, plant genotype, soil properties, formulation, colonization capacity, and interactions with indigenous microorganisms. Therefore, bacterial inoculants should be selected according to experimentally validated functions and ecological compatibility rather than taxonomic identity alone [71,72,73,77].

#### 3.1.2. Fungal Inoculants and Fungus-Mediated Interactions

Fungal inoculants remain an important complementary component of soil microbiome management. Boutasknit et al. (2021) demonstrated that the combined application of indigenous arbuscular mycorrhizal fungi and green-waste compost improved drought tolerance in carob more effectively than the individual treatments, indicating that inoculant performance depends on the ecological conditions supporting microbial establishment [78]. *Claroideoglomus etunicatum* altered rhizosphere community functions and improved maize performance under combined metal, salinity, and boron stresses, whereas *Entrophospora etunicata* enhanced the accumulation of bioactive compounds in *Capsicum chinense* [80,81]. Collectively, these findings show that mycorrhizal fungi can contribute to nutrient transfer, stress tolerance, and crop quality. However, their effects remain dependent on fungal identity, host species, soil conditions, and compatibility with resident microbial communities [78,79,82].

#### 3.1.3. Synthetic Bacterial Communities

Recent microbiome engineering strategies increasingly move beyond single-strain inoculants toward function-oriented synthetic bacterial communities. In these systems, strains are selected according to complementary traits such as nutrient mobilization, root colonization, stress mitigation, pathogen suppression, and metabolic cross-feeding rather than simply being combined on the basis of taxonomic diversity [70,71].

A representative example is the soybean-derived bacterial SynCom described under nitrogen-deficient conditions. The community was reconstructed from a low-nitrogen-enriched bacterial cluster and reproduced the plant growth-promoting effect identified in the original microbiome, thereby providing causal evidence that bacterial community succession can contribute to crop productivity under nutrient limitation [43].

Recent evidence also demonstrates that bacterial SynComs may generate functions that are absent or weaker in individual strains. A four-member Gram-positive bacterial SynCom containing *Bacillus* and related taxa provided stronger protection of pepper plants than individual bacterial inoculations under field conditions. The complete community produced the volatile compound 1-nonanol and activated plant defense responses, demonstrating that microbial interactions can generate emergent metabolites and biocontrol functions [83].

However, increasing the number of strains does not automatically improve SynCom performance. Community stability depends on strain compatibility, initial abundance ratios, metabolic interactions, functional redundancy, colonization capacity, and competition with indigenous microorganisms. Consequently, rational SynCom design should combine genome- and trait-based strain selection with pairwise compatibility testing, community-level validation, and field assessment [71,84]. The principal functional categories, representative bacterial genera, underlying mechanisms, and relevance of beneficial bacteria to soil microbiome engineering are summarized in Table 3.

### 3.2. Microbiome Transplantation and Rhizosphere Engineering

Microbiome transplantation and rhizosphere steering are primarily forms of microbiome manipulation because they modify an existing microbial community or its ecological environment rather than constructing a new community through rational design. Microbiome transplantation aims to transfer an entire functional microbial community rather than individual microorganisms, whereas rhizosphere engineering focuses on modifying root-associated environments to favor the establishment of beneficial microbial assemblages. These approaches are based on the recognition that microbiome functions often emerge from complex interactions among microorganisms, metabolites, and environmental conditions rather than from the activity of single taxa alone [90,91]. Consequently, transplantation and rhizosphere engineering have gained increasing attention as alternatives to conventional inoculation strategies, particularly in cases where single-strain applications show inconsistent performance under field conditions.

Hernández-Álvarez et al. (2022) demonstrated that microbiome transplantation can transfer adaptive functions as well as microbial communities [92]. Using squash rhizosphere microbiomes from arid and humid environments, the study showed that transplanted communities retained distinct taxonomic and functional characteristics under greenhouse conditions. Notably, arid-derived microbiomes remained enriched in traits associated with osmotic regulation and stress tolerance, indicating the transfer of ecological memory.

Rhizosphere engineering can also be achieved indirectly through host-mediated selection. A common-garden study involving European tree species with contrasting mycorrhizal associations demonstrated that plant identity and mycorrhizal type strongly influence soil microbial biomass and metabolic activity. Soil pH and C:N ratio emerged as major factors regulating these responses. Although not a transplantation experiment, the study showed that manipulation of plant traits and litter chemistry can steer rhizosphere microbial communities by altering the ecological conditions in which they develop [93].

The concept of rhizosphere engineering has expanded toward improving crop nutritional quality. Arbuscular mycorrhizal symbiosis can enhance the transport and accumulation of ergothioneine in plant tissues, indicating that targeted root–fungus interactions may influence the transfer of specific soil-derived metabolites to crops [94]. Fungal hyphal networks can also serve as physical pathways for the movement of microorganisms and metabolites, thereby improving microbial dispersal and ecological connectivity within the rhizosphere [95]. The physiological benefits of rhizosphere manipulation are particularly evident under water-limited conditions. In Caatinga passion fruit, mycorrhizal colonization improved photosynthetic performance, water-use efficiency, and stress-responsive gene expression [96]. Collectively, these studies show that fungal-mediated rhizosphere manipulation can influence nutrient transfer, microbial connectivity, and plant stress responses, although its effectiveness remains dependent on host species and environmental conditions.

### 3.3. CRISPR, Synthetic Biology, and Engineered Microbes

In contrast to the manipulation-based approaches described above, CRISPR, synthetic biology, and engineered microorganisms represent microbiome engineering in the stricter sense because they involve deliberate functional design, genetic modification, or programmable reassembly. The integration of clustered regularly interspaced short palindromic repeats (CRISPR) technologies, synthetic biology, and engineered microorganisms has shifted soil microbiome engineering from microbial selection toward programmable functional design [97,98]. Unlike conventional inoculation approaches, these technologies seek to control specific microbial traits, including nutrient exchange, stress-responsive gene expression, metabolic pathways, and host–microbe interactions. However, the deployment of engineered microorganisms in soil remains challenging because environmental conditions, ecological competition, and biosafety considerations can strongly influence their performance outside laboratory settings.

One of the most important targets for microbial engineering is biological nitrogen fixation. Ryu et al. (2020) investigated regulatory mechanisms controlling nitrogen fixation in *Azorhizobium caulinodans*, a bacterium capable of colonizing cereal crops such as rice and wheat [99]. Although the bacterium successfully established itself as an endophyte, nitrogenase activity remained limited within cereal roots due to regulatory constraints. These findings demonstrated that successful engineering requires not only microbial colonization but also precise control of gene expression within the target host environment.

A major challenge in synthetic biology is the development of efficient microbial chassis that can be readily modified and deployed in soil ecosystems. Recent advances in *Pseudomonas putida* KT2440 have addressed this limitation through the development of simplified CRISPR/Cas9 genome-editing systems that enable rapid and iterative genetic modification. Improved editing efficiency and plasmid-curing strategies substantially reduce the time required to construct engineered strains, thereby facilitating the development of microorganisms with customized rhizosphere functions [100].

Particular attention has been given to *Pseudomonas putida* KT2440 because of its metabolic versatility, environmental relevance, and established biosafety profile. The strain is considered one of the most promising chassis organisms for future microbiome engineering applications, providing a platform for the development of rhizosphere-responsive genetic circuits, metabolite exchange modules, and biological containment systems. Although large-scale agricultural deployment remains limited, advances in chassis engineering are laying the foundation for more predictable and controllable microbiome interventions [100,101].

Current applications of engineered microorganisms remain largely focused on nutrient acquisition processes, particularly nitrogen fixation and nutrient exchange pathways. These functions are attractive engineering targets because they provide measurable agronomic benefits and are regulated by relatively well-characterized genetic networks. At the same time, they highlight the challenges associated with field deployment, including oxygen sensitivity, host-dependent regulation, metabolic costs, and competition with indigenous microbial communities. Consequently, the transition from successfully engineered laboratory strains to reliable field-performing microbial systems remains one of the major challenges in soil microbiome engineering [102,103].

Recent advances in CRISPR technologies, synthetic biology, and chassis development have significantly expanded the potential for precision microbiome engineering. Nevertheless, achieving stable performance under field conditions will require not only sophisticated genetic tools but also a deeper understanding of microbial ecology, host interactions, and environmental constraints that shape engineered microbial functions in agricultural soils [99,100,101,102,103].

### 3.4. Critical Comparison of Soil Microbiome Intervention Strategies

The strategies discussed in this section span a continuum from ecological manipulation to rational engineering. Inoculation, microbiome transplantation, and rhizosphere steering primarily modify existing microbial communities, whereas rationally designed SynComs and genetically engineered microorganisms provide progressively greater compositional and functional control [90,91,97]. Single-strain inoculants are relatively straightforward to formulate and standardize, but their field performance is frequently limited by poor establishment, insufficient persistence, and competition with resident microorganisms [18,72]. Whole-community transplantation preserves ecological interactions and functional diversity, although donor-community composition and performance may be difficult to reproduce consistently across recipient soils and host plants [90,91,92]. SynComs occupy an intermediate position by combining defined composition with functional complementarity, but their behavior remains sensitive to strain compatibility, community succession, initial abundance ratios, and environmental conditions [70,71,89]. Engineered microorganisms offer greater functional precision, although their deployment is constrained by host-dependent gene regulation, genetic stability, metabolic burden, biosafety, and ecological containment requirements [99,100,101].

Consequently, no single intervention strategy is universally superior. The most appropriate approach depends on the intended function, crop genotype, soil properties, resident microbiome, and required duration of activity [71,72]. Functional complementarity, colonization capacity, and compatibility with native communities may be more important for field performance than simply increasing the number of strains included in an inoculant [70,71,89]. A central unresolved question is whether agricultural microbiomes should be directly modified through inoculation, transplantation, designed communities, or engineered microorganisms, or indirectly steered by modifying plant traits, root exudation, and soil conditions [53,90,97]. Comparative field experiments using standardized endpoints are therefore required to determine which strategies provide stable functions under realistic agricultural conditions [71,72]. The principal advantages, limitations, and supporting evidence for these intervention strategies are critically compared in Table 4.

## 4. Multi-Omics and AI Approaches in Soil Microbiome Research

### 4.1. Metagenomics and Functional Omics

Scientifically, metagenomics and functional omics are important because they resolve different layers of the same problem: who is present, what microorganisms are capable of doing, which functions are actively expressed, and how these activities change under ecologically relevant stress [104]. In soils, where dormant taxa are common and environmental filtering is strong, this distinction is crucial [105].

**Figure 3:**
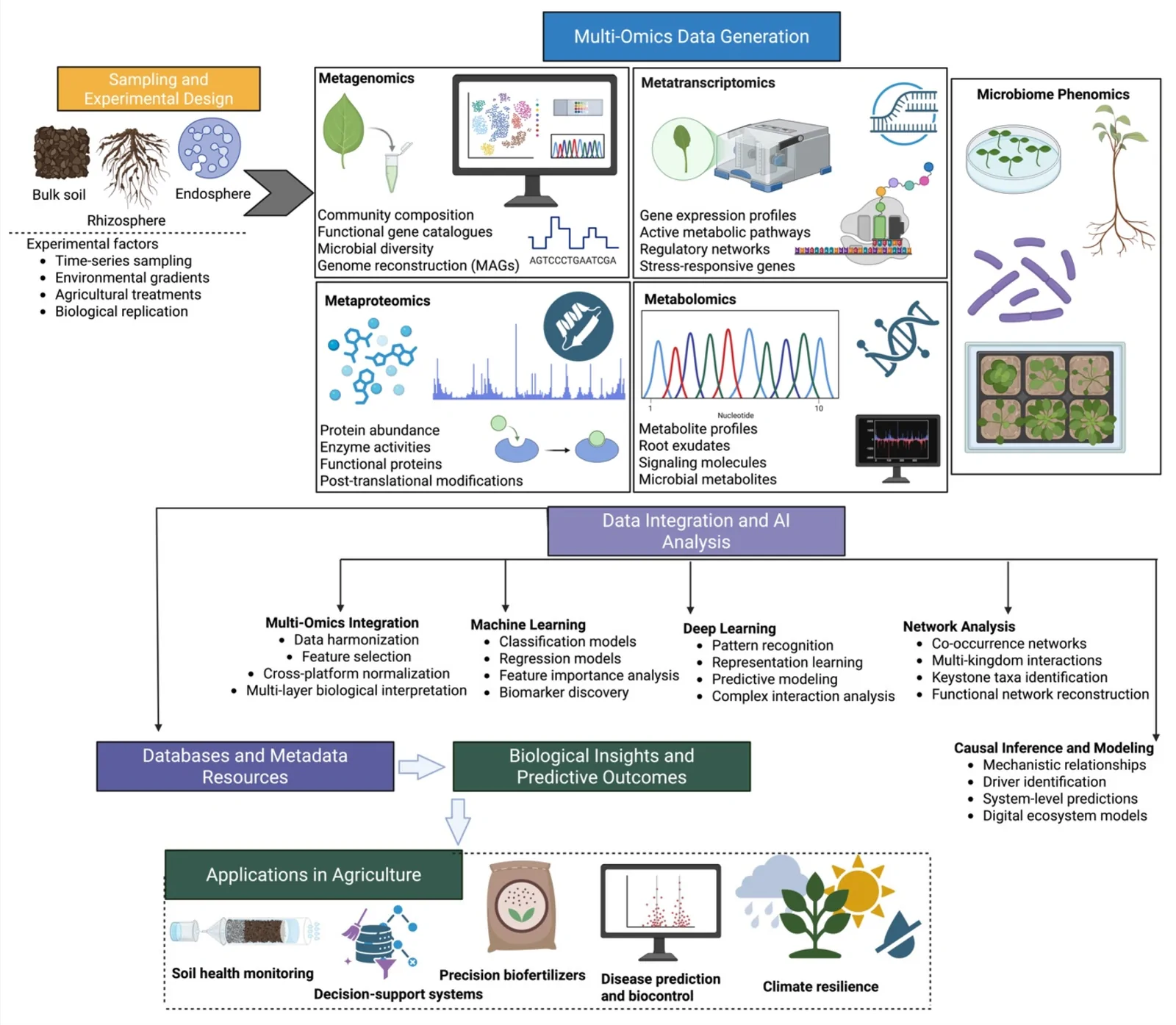
Multi-omics and AI approaches in soil microbiome research. Multi-omics technologies, including metagenomics, metatranscriptomics, metaproteomics, metabolomics, and phenomics, generate complementary layers of biological information. Integration of these datasets through ML, deep learning, network analysis, and causal modeling enables the identification of microbial functions, ecological interactions, biomarkers, and predictive outcomes relevant to soil health, crop productivity, and sustainable agriculture. Arrows indicate the direction of data and information flow, from sampling and experimental design through multi-omics data generation and AI-based integration to biological insights, predictive outcomes, and agricultural applications. Created in BioRender, https://BioRender.com/2jiilci (access on 23 July 2026).

Metagenomics primarily reveals the functional potential of microbial communities, whereas metatranscriptomics, metaproteomics, lipidomics, metabolomics, and artificial intelligence-assisted data integration provide stronger evidence of active ecological processes. As summarized in Figure 3, multi-omics technologies generate complementary layers of biological information that can be integrated through machine learning, network analysis, and causal modelling. This integrated workflow enables the identification of microbial functions, ecological interactions, biomarkers, and predictive outcomes relevant to soil health, crop productivity, and sustainable agriculture. McClure et al. (2020) developed a stable reduced-complexity soil consortium enriched on chitin from arid grassland soil [106]. The key methodological innovation was not merely simplification, but preservation of ecologically meaningful diversity: the consortium retained 30–50 operational taxonomic units (OTUs) spanning major soil phyla and could be cryopreserved and revived without major compositional drift. Co-abundance analysis then identified likely keystone taxa such as *Mycobacterium*, *Rhodococcus*, and *Rhizobiales*. This was important because it created a tractable platform for experimentally testing multi-omic hypotheses that would be nearly impossible in whole soil.

In the second study, Naylor et al. (2020), the same general strategy was pushed further by partitioning a complex soil microbiome into low complexity “functional modules” using selective enrichments that varied carbon sources, redox conditions, antibiotics, and stress regimes [107]. Amplicon sequencing and metatranscriptomics showed that restrictive conditions generated more distinct, richer, but less reproducible modules, whereas simple substrates yielded lower-richness yet more repeatable communities. Roughly 27% of unique taxa present in the liquid soil extract control were recovered across these modules. The contribution of this study was conceptual as much as technical: it showed that decomposition and other soil functions can be studied through a modular, experimentally decomposed microbiome rather than only through whole-community correlative analyses.

A different scale of functional-omic ambition appears in the prairie virome study of Wu et al. (2021) [108]. In Communications Biology, the authors combined metagenomics, metatranscriptomics, and metaproteomics to identify active DNA and RNA viruses in native prairie soils exposed to extreme moisture differences. The methodological significance was the triangulation of activity: viral sequences were not merely detected in DNA; they were shown to be transcribed, and selected transcripts were validated at the protein level. Higher moisture increased transcription of a subset of DNA viruses, while wet soils also contained more diverse RNA viral signals, especially among leviviruses. This study materially advanced soil functional omics by demonstrating that viral activity can be monitored as a living component of the soil metaphenome rather than as passive sequence residue.

The related study Wu et al. (2021) in mBio extended soil viromics from activity to environmental structuring [109]. Using three ultra-deep grassland soil metagenomes from sites spanning low, intermediate, and high historical precipitation, the authors reconstructed 2631 viral contigs, including complete viral genomes, and compared viral abundance, host linkage, lifestyle markers, and auxiliary metabolic genes. Drier Washington soils showed higher viral and host diversity, more lysogeny-associated markers, fewer CRISPR spacer hits, and more putative auxiliary metabolic genes (AMGs) related to carbon metabolism and energy acquisition. Structural equation modeling suggested that historical precipitation shaped viral life cycle and AMG selection. The contribution of this paper lies in showing that soil climate history is written into viral functional repertoires, with direct implications for nutrient cycling and drought adaptation.

A shorter-timescale but equally revealing study is Couvillion et al., 2023, which tracked lipidomic and metabolomic changes during drying and within the first 3 h after wet-up [110]. Dry soil replaced glycerophospholipids with phosphorus-free lipids, limited expensive osmolyte accumulation, and enriched ceramides and long-chain polyunsaturated lipids interpreted as fungal drought-tolerance markers. Rewetting rapidly increased bacterial-associated glycerophospholipids, triacylglycerols, and polar metabolites consistent with metabolic recovery. This work is important because it shows that the soil lipidome is not a generic stress readout; it is a temporally resolved window into nutrient limitation, membrane remodeling, and taxon-specific recovery trajectories.

### 4.2. Systems Biology and Microbial Network Analysis

The scientific relevance of systems biology in soils is that soil function is almost never the product of a single taxon. It emerges from interacting guilds, trophic dependencies, viral controls, and environmentally conditioned phenotypes [111]. For this reason, the strongest network studies are those that move beyond simple co-occurrence to infer mechanistic constraints: substrate flow, phage–host linkage, phenotype switching, or network centrality under perturbation [112].

A foundational systems-level study is Zegeye et al., 2019, who followed 21 weeks of succession on chitin and N-acetylglucosamine in both soil matrices and liquid medium [113]. Their core question was whether initial richness governs stabilization. The answer was yes, but only in interaction with physical context: lower starting richness accelerated convergence, and consortia selected in soil diverged substantially from those selected in homogenized liquid despite sharing inoculum and substrate. This work contributed a critical principle that later network studies repeatedly confirmed physical structure is not a background variable but part of the interaction network itself. McClure et al., 2020 provided a complementary systems platform by analyzing interspecies interactions in a stable reduced-complexity consortium derived from soil [106]. Pairwise cocultivation and soil perturbation experiments, combined with co-abundance inference, highlighted taxa likely to govern community behavior under chitin degradation. Its contribution to network analysis lies in its experimental tractability: unlike many purely correlation-based networks, this system could be perturbed, stored, revived, and reanalyzed, making network hypotheses testable rather than merely inferential. The clearest phenotype-to-network link in the recent literature appears in McClure et al., 2022 [114]. Working with a simplified chitin-degrading consortium, the authors showed that some species behaved as primary degraders while others outperformed them by exploiting released breakdown products; more importantly, whether a species behaved as a primary degrader depended strongly on community membership. In other words, phenotype was not fixed at the isolate level but was community responsive. This is a major systems-biology contribution because it shows that network position and resource phenotype must be inferred jointly, not sequentially.

A different type of network inference was delivered by Wu et al. (2023) project metadata on Hi-C metagenomics under dry versus wet soils [115]. By using high-throughput chromosomal confirmation capture, the authors directly linked phages to hosts rather than inferring interactions from covariance alone. Wet soils showed higher transcription of host-associated phages, while desiccated soils had higher richness of linked phages and higher average viral copies per host, suggesting a shift toward more prevalent lysogeny after drying. Infection-network analysis further showed that moisture altered which hosts were central in bacterial co-occurrence networks. Methodologically, this is important because it demonstrates how structural genomics can turn vague “viral influence” into explicit infection topology.

### 4.3. Artificial Intelligence and Machine Learning

AI and ML are becoming increasingly important in soil microbiome research because microbial datasets are often high-dimensional, sparse, compositional, and strongly influenced by environmental variables [116,117]. Although conventional statistical approaches remain valuable for hypothesis testing, they are frequently insufficient for predicting crop performance, identifying microbial indicators, integrating multi-omics datasets, or modeling complex nonlinear interactions among soil properties, microbial communities, and plant traits [118]. Consequently, AI and ML have emerged as powerful tools that bridge descriptive microbiome characterization and predictive agroecosystem management [119].

Aghdam et al. (2024) provided one of the clearest examples of microbiome-based prediction by evaluating whether potato yield and disease outcomes could be inferred from soil microbiome data [120]. Comparing random forest and Bayesian neural network models, the authors demonstrated that prediction accuracy was strongly influenced by preprocessing strategies, taxonomic resolution, metadata integration, and label quality. The study highlighted that successful microbiome prediction depends not only on algorithm selection but also on biologically informed data preparation and interpretation.

Beyond prediction, recent efforts have focused on improving the interpretability of ML outputs. Using explainable AI approaches based on Shapley Additive Explanation (SHAP) values, Hagen et al. (2024) identified microbial taxa associated with drought-induced shifts in soil communities [121]. Importantly, many of the microbial indicators detected by ML overlapped with results obtained through conventional differential abundance analyses, demonstrating that explainable AI can complement rather than replace traditional microbiome statistics. This work represents an important step toward making ML-derived predictions biologically meaningful and mechanistically interpretable.

ML has also proven useful for disentangling complex management effects within soil profiles. By integrating 16S/ITS sequencing, co-occurrence networks, microbial assembly models, and feature attribution analyses, recent work revealed that fertilizer source was the dominant factor shaping microbial communities throughout the soil profile, whereas tillage effects were largely confined to surface layers. These findings demonstrate the capacity of ML approaches to detect depth-dependent ecological patterns that may be overlooked when soils are analyzed as a single homogeneous system [45,122].

Another emerging direction involves the integration of microbiome data with remote sensing technologies. In wheat, barley, and maize production systems, fungal community composition was linked with crop health indicators derived from multispectral satellite imagery through a two-stage ML framework. After correcting vegetation indices for abiotic influences, residual variation in crop performance remained associated with specific fungal community structures. This approach demonstrates how AI can connect belowground microbial information with large-scale crop monitoring and precision agriculture applications [123].

Machine learning approaches have also been applied to connect soil microbiome patterns with plant defense and ecosystem-service outcomes. In organically managed agricultural soils, ML-based analysis linked management-associated microbial shifts with plant defense signaling and reduced aphid pressure [47,124]. More recently, Aouabed et al. (2026) compared traditional ML algorithms with deep learning models for predicting bacterial and fungal community composition from environmental variables [125]. Interestingly, random forest and k-nearest-neighbor approaches frequently outperformed multilayer perceptron models, particularly when datasets were relatively small. The study emphasizes that increasing model complexity does not necessarily improve predictive performance and that simpler, more interpretable algorithms may often be better suited for soil microbiome datasets.

#### Practical Implementation, Public Data Resources, and Current Limitations

Practical implementation of AI-based soil microbiome prediction requires more than fitting an algorithm to microbial abundance tables. A deployable workflow should integrate bacterial and fungal community profiles with soil physicochemical properties, weather conditions, management history, host genotype, and clearly defined agronomic outcomes. In the potato study discussed above, microbiome data showed predictive value for some disease outcomes but were insufficient for reliable yield prediction when used alone. Prediction improved when microbial information was combined with environmental and soil variables, demonstrating that microbiome-based models should be developed as integrated decision-support systems rather than as stand-alone taxonomic predictors [120].

Model evaluation is also critical for practical implementation. Training, validation, and test datasets should be separated according to field, geographic location, growing season, or year whenever possible, because random division of closely related samples may overestimate model performance. Feature interpretation methods such as Shapley Additive Explanations (SHAP) can identify microbial taxa that contribute strongly to predictions; however, such taxa remain candidate biomarkers until their functions are confirmed through microbial isolation, inoculation, SynCom reconstruction, or other perturbation experiments [121].

Several publicly accessible resources can support model development, cross-study comparison, and external validation. The Sequence Read Archive (SRA) provides access to raw high-throughput sequencing data from metagenomic and environmental studies, whereas MGnify provides harmonized taxonomic and functional analyses generated through standardized pipelines. Qiita supports microbiome study management, standardized metadata organization, and cross-study meta-analysis [126,127,128]. These resources can increase sample size and environmental coverage, but only datasets with sufficiently complete information on sample type, crop, location, soil properties, management practices, and sequencing methodology should be combined for predictive modelling.

Despite their potential, current AI-based microbiome models face several limitations. Microbiome datasets commonly contain many more microbial features than samples and are characterized by sparsity, compositionality, class imbalance, and strong sensitivity to preprocessing choices [117,118]. Differences in sampling procedures, DNA extraction, sequencing platforms, taxonomic databases, and bioinformatic pipelines can introduce batch effects that models may incorrectly interpret as biological signals. Moreover, models trained in one crop, soil type, or geographic region may show reduced performance when transferred to other environments because microbial associations are strongly context dependent [117,118].

Increasing model complexity does not necessarily overcome these limitations. Comparisons of traditional ML and deep learning approaches indicate that random forest and k-nearest-neighbor models can outperform multilayer perceptrons when sample sizes are limited [125]. Therefore, algorithm selection should be based on external predictive performance, interpretability, computational requirements, and the intended agricultural application rather than on model complexity alone. Prospective validation across multiple locations and growing seasons remains necessary before AI-based microbiome predictions can be incorporated into routine crop or soil management (Table 5).

### 4.4. Precision Agriculture and Smart Soil Monitoring

Recent advances have shifted soil microbiome studies from static end-point assessments toward dynamic, field-scale analyses of microbial activity and distribution [129,130]. Naylor et al. (2023) provided a compelling example of precision microbiome monitoring through a field experiment incorporating four irrigation regimes and three soil depths [131]. By integrating microbial community composition, transcriptomics, and metabolomics across bulk soil and rhizosphere compartments, the authors demonstrated that microbiome responses to moisture differed substantially between surface and deeper soil layers. These findings highlight the importance of depth-specific monitoring strategies and suggest that microbiome-based indicators cannot be assumed to respond uniformly throughout the soil profile.

The value of depth-resolved monitoring was further supported by studies linking microbiome activity with soil carbon dynamics. By combining multi-omics analyses with detailed carbon chemistry measurements in calcareous soils, distinct functional signatures were identified between surface and deep soil horizons. Notably, oxidable carbon showed stronger associations with microbial metabolites and proteins than total soil organic carbon, suggesting that dynamic biochemical indicators may provide more informative measures of biologically active carbon turnover for future monitoring systems [132].

Smart soil monitoring is also moving toward real-time assessment of microbial processes. Using ^13^C-labelled glucose and isotopic discrimination of respiratory CO_2_ fluxes immediately following soil rewetting, recent work demonstrated that microorganisms initially utilize pre-existing carbon pools before metabolizing newly available substrates. The study revealed measurable functional responses within minutes after moisture change, illustrating the potential of isotope-based approaches for high-resolution monitoring of microbial activity and resource utilization [133].

Advances in microscale visualization technologies are providing an additional layer of insight into rhizosphere processes. Using soil micromodels combined with mass spectrometry imaging, X-ray fluorescence, and X-ray absorption spectroscopy, Bhattacharjee et al. (2023) visualized fungal-mediated mineral weathering and potassium mobilization under soil-like conditions [134]. Spatial distributions of organic acids and mineral-derived nutrients were mapped directly within fungal networks, demonstrating how microscale imaging platforms can generate mechanistic datasets for calibrating sensor technologies and computational rhizosphere models.

Precision monitoring is increasingly extending beyond field and laboratory scales toward landscape-level assessments. A recent global analysis of arbuscular mycorrhizal fungal networks employed ML to estimate fungal connectivity across diverse biomes. The resulting maps provide a large-scale biological baseline for evaluating land-use change, ecosystem restoration, and soil health status. Although not designed for immediate field management, this work illustrates how predictive modelling, and large environmental datasets can support future soil monitoring frameworks and ecosystem-scale decision making [135].

Despite their complementary strengths, these technologies address different levels of microbiome organization and should not be interpreted as interchangeable sources of evidence. Amplicon sequencing primarily describes community composition, metagenomics identifies functional potential, and metatranscriptomics, proteomics, or metabolomics provide progressively stronger evidence of active biological processes. Network analysis and machine learning models can identify important associations and predictive features, but these outputs do not establish direct ecological interactions or causality without experimental validation. Moreover, differences in sampling, sequencing depth, data preprocessing, and environmental metadata can generate apparently contradictory results across studies. The most reliable framework therefore combines multi-omics prediction with perturbation experiments, microbial isolation, SynCom reconstruction, or microbiome transplantation. A continuing methodological challenge is to achieve this level of validation while retaining the ecological complexity of field soils.

## 5. Agricultural and Environmental Applications

### 5.1. Sustainable Crop Production and Nutrient Management

Sustainable crop production and nutrient management are closely linked to the functioning of the soil microbiome, which regulates nutrient mobilization, mineralization, retention, and loss within agricultural ecosystems [135,136]. From a microbiome perspective, sustainable intensification depends not only on nutrient inputs but also on maintaining rhizosphere communities that support nutrient cycling, preserve soil structure, and sustain long-term soil fertility under continuous cultivation [137].

Arafat et al. (2020) provided one of the clearest examples of microbiome-associated productivity decline in perennial cropping systems [138]. Investigating long-term tea plantations, the authors demonstrated that the accumulation of plant-derived polyphenols was accompanied by pronounced shifts in soil microbial communities. Their findings suggested that soil sickness arises from the combined effects of unfavorable biochemical conditions and microbial dysbiosis rather than nutrient depletion alone. This work highlights the importance of maintaining rhizosphere ecological balance for sustaining long-term productivity.

Similar patterns have been reported in monoculture systems. Continuous sugarcane cultivation was shown to alter microbial community composition, reduce microbial activity, increase soil acidification, and ultimately weaken soil fertility. These observations emphasize that simplified cropping systems can simultaneously affect soil chemistry and microbial functions, thereby reducing the efficiency of nutrient cycling processes. Consequently, diversified management practices may be required to restore microbiome functions that support sustainable nutrient turnover [139].

Management interventions that directly influence microbial communities can provide an alternative strategy for improving soil fertility. For example, straw retention in sugarcane-associated systems promoted beneficial shifts in fungal community composition and functional potential. Because fungi play key roles in residue decomposition, nutrient mineralization, and aggregate formation, these findings support crop residue management as an effective tool for microbiome-based enhancement of soil fertility and nutrient availability [140].

Microbial contributions to crop productivity are not limited to nutrient transformations alone. Le Gall et al. (2021) demonstrated that increased microbial exopolysaccharide production under drought conditions improved soil water retention and enhanced tomato performance [141]. Although primarily investigated in the context of water stress, these results are also relevant to nutrient management because improved soil moisture facilitates nutrient diffusion, root–soil contact, and nutrient uptake efficiency. The study illustrates how microbiome-mediated modifications of soil physical properties can indirectly influence crop nutrition and productivity.

More recently, attention has turned toward the influence of crop establishment practices on rhizosphere assembly. Investigation of sugarcane propagated through different methods revealed significant differences in root metabolite profiles and associated microbial communities. These findings suggest that propagation strategy can shape rhizosphere microbiome development from the earliest stages of crop establishment, ultimately affecting nutrient acquisition and long-term soil functioning. Such observations broaden the concept of nutrient management by highlighting the role of plant-associated factors in microbiome assembly [142].

Current evidence indicates that sustainable crop production depends on maintaining functionally diverse microbial communities capable of supporting nutrient cycling, soil structure, and plant health. Long-term monoculture, soil sickness, and degradation of microbial functions can reduce productivity even when nutrient inputs remain adequate. Conversely, practices such as residue retention, improved water management, and microbiome-conscious crop establishment can enhance nutrient-use efficiency by promoting beneficial soil microbial processes [138,139,140,141,142].

### 5.2. Microbiome-Based Biocontrol Strategies

Microbiome-based biocontrol has emerged as an important alternative to conventional pesticide-dependent disease management because it relies on ecological suppression rather than direct pathogen elimination [143,144]. Disease control in these systems can result from multiple mechanisms, including competitive exclusion, antimicrobial metabolite production, induced systemic resistance, trophic interactions, and the establishment of beneficial microbial communities within the rhizosphere. Consequently, recent research increasingly views disease suppression as a property of the soil ecosystem rather than the activity of a single antagonistic organism.

Several field and experimental studies provide concrete examples of microbiome-based biocontrol strategies. One approach is to manage resident suppressive communities by modifying soil conditions. Soil acidification weakened the natural capacity of indigenous microbiomes to suppress *Fusarium* infection, demonstrating that biocontrol functions depend strongly on environmental context [46]. Conversely, soil solarization and reductive soil disinfestation reduced pathogen pressure while promoting the reassembly of bacterial and fungal communities associated with suppressive soil conditions [145,146]. Host-mediated recruitment represents another strategy: in tomato, pathogen-derived fusaric acid altered root exudation and influenced the recruitment of the disease-suppressive bacterium *Sphingomonas* sp. Sm12, with resistant cultivars assembling more suppressive rhizosphere communities than susceptible cultivars [56].

Targeted microbial interventions provide a complementary route to microbiome-based biocontrol. *Lysobacter capsici* AZ78 produces antimicrobial metabolites with activity against plant pathogens, illustrating how functionally characterized bacterial inoculants can be selected for predictable biocontrol mechanisms [79]. A four-member Gram-positive bacterial SynCom also provided stronger protection of pepper plants against aphid infestation than individual strains through community-dependent production of 1-nonanol and activation of plant defense responses [83]. At the community level, biofertilizer application enriched predatory protists, particularly *Cercomonas*, together with genes involved in secondary-metabolite biosynthesis, thereby contributing to soil disease suppression through trophic regulation rather than direct bacterial antagonism alone [49]. These examples show that microbiome-based biocontrol can involve resident-community steering, host-mediated recruitment, targeted inoculation, designed microbial communities, and food-web regulation.

Among microbial antagonists, *Pochonia chlamydosporia* represents a particularly interesting example because it combines pest suppression with plant growth-promoting functions. Studies have demonstrated that this fungus produces metabolites associated with nematicidal activity while simultaneously functioning as a rhizosphere-competent endophyte. In addition, its ability to mobilize phosphorus and produce organic acids may contribute to improved nutrient availability. These multifunctional traits illustrate how a single microorganism can support both plant protection and nutrient acquisition, blurring the traditional distinction between biocontrol and biofertilizer agents [147].

Effective biological control also depends on the long-term persistence of beneficial organisms within soil ecosystems. Investigations of entomopathogenic fungi have shown that successful suppression is influenced not only by antagonistic activity but also by the ability of microbial populations to establish, persist, and interact with indigenous soil communities under field conditions. These observations emphasize that ecological stability is a key determinant of long-term biocontrol performance [148].

The development of highly sensitive pathogen-detection systems further supports microbiome-based disease management. Molecular approaches capable of detecting soil- and water-borne pathogens in agricultural drainage systems have improved surveillance and early warning capabilities, thereby strengthening integrated disease management strategies at field and landscape scales [149].

Similarly, advances in automated soil pest monitoring are improving the implementation of biological control programs. Robotic extraction and quantification systems provide more accurate assessments of soil pest populations, facilitating the evaluation of suppressive potential and the optimization of microbiome-based interventions. Such technologies are particularly important because the effectiveness of biological suppression often depends on pathogen density, environmental conditions, and site-specific ecological factors [150].

### 5.3. Climate-Smart and Regenerative Agriculture

Climate-smart and regenerative agriculture are microbiologically significant because the resilience of agricultural systems depends not only on crop genetics or climatic conditions but also on the capacity of soil microbial communities to maintain nutrient cycling, stabilize soil structure, improve water retention, and recover following environmental disturbance [151,152]. Regenerative management practices influence the ecological filters that shape microbial community assembly, while climate-related stresses increasingly favor microorganisms capable of osmotic adjustment, exopolysaccharide production, and stress-tolerant nutrient transformation [153].

Microbiome-based interventions can improve crop tolerance to drought, salinity, nutrient limitation, and combined stresses. Beneficial bacteria such as *Aeromonas*, *Pseudomonas*, and *Rhizobium* enhance stress resistance through effective host-signal responses, root colonization, and stress-adaptive functions [64,65,66]. Broader protection may be achieved using SynComs, arbuscular mycorrhizal fungi, and integrated microbial–soil amendment strategies, which can enhance plant performance and microbial community stability under multiple environmental stresses [68,80,82].

One of the strongest demonstrations of climate-adapted microbiome functionality was provided through rhizosphere transplantation experiments involving squash microbiomes originating from historically arid and humid environments. Even after transfer to a common greenhouse environment, arid-derived communities retained distinct taxonomic and functional characteristics, including enrichment of genes associated with osmotic regulation, oxidative stress tolerance, compatible solute synthesis, and protein stabilization. These findings suggest that microbiomes can preserve ecological memory of past environmental conditions and potentially transfer climate-adaptive functions to new cultivation systems [85].

Evidence supporting regenerative agriculture has also emerged from large-scale quantitative syntheses. A second-order meta-analysis based on 184 meta-analyses and 6741 effect sizes showed that agricultural diversification practices significantly improved biodiversity, soil quality, carbon sequestration, and financial profitability over time. These findings suggest that diversification-based regenerative management promotes ecological conditions that support long-term soil health and resilient microbial communities [154].

The successful implementation of regenerative agriculture increasingly depends on data-driven monitoring systems capable of tracking changes in soil function. Recent decision-support frameworks integrating management history, extreme-weather records, and soil organic carbon predictions have demonstrated that diversified farming practices can mitigate the negative effects of intensive tillage, while compost application can buffer carbon losses under adverse climatic conditions. Such approaches highlight the growing importance of combining microbiome-informed management with predictive monitoring tools to support long-term soil resilience [155].

Climate-smart soil management is also becoming increasingly dependent on spatially explicit environmental diagnostics. Machine learning models developed for coastal agricultural regions have successfully predicted soil salinity using field observations and satellite-derived indicators. Although these studies do not directly analyze microbial communities, they identify environmental stress gradients that strongly influence rhizosphere assembly and microbial functioning. Consequently, high-resolution monitoring systems can help identify areas where microbiome-based interventions may be most beneficial for improving stress tolerance and sustaining productivity under changing climatic conditions [156].

### 5.4. Soil Restoration, Carbon Sequestration, and Ecosystem Recovery

Soil restoration and carbon sequestration have become central objectives of sustainable agriculture because soil degradation, biodiversity loss, and declining organic matter threaten both agricultural productivity and ecosystem stability [157,158]. The soil microbiome plays a fundamental role in these processes by regulating organic matter decomposition, carbon stabilization, nutrient cycling, aggregate formation, and plant establishment during ecosystem recovery [159]. Consequently, restoration success is increasingly viewed not only as the recovery of soil physicochemical properties but also as the re-establishment of functional microbial communities capable of supporting long-term ecosystem resilience [160].

One of the most important advances in recent years has been the recognition that soil microorganisms directly influence the fate of carbon inputs entering agricultural and natural ecosystems. Long-term field studies have shown that microbial community composition and functional diversity determine whether plant-derived carbon is rapidly mineralized and released as carbon dioxide or incorporated into stable soil organic matter pools. These findings highlight the central role of microbial processes in regulating carbon sequestration efficiency and long-term soil fertility [161].

The importance of microbial diversity for ecosystem recovery has also been demonstrated in studies of degraded and restored soils. Investigations comparing restored and non-restored ecosystems consistently report increases in microbial biomass, community complexity, and functional gene diversity following restoration interventions. Enhanced microbial diversity is frequently associated with improved nutrient retention, greater ecosystem stability, and accelerated recovery of soil ecological functions, indicating that microbial communities can serve as sensitive indicators of restoration progress [162].

Organic amendments represent one of the most widely applied microbiome-mediated restoration strategies. Applications of compost, biochar, and organic residues have been shown to increase microbial abundance, stimulate beneficial functional groups, and promote the accumulation of soil organic carbon. In addition to supplying carbon substrates, these amendments improve soil structure and create favorable microhabitats for microbial colonization. As a result, restored soils often exhibit enhanced nutrient cycling capacity, greater aggregate stability, and improved resistance to environmental stress [163].

The role of microorganisms in aggregate formation has attracted particular attention because soil aggregates physically protect organic matter from decomposition and contribute to long-term carbon storage. Microbial exopolysaccharides, fungal hyphae, and root-associated microbial networks act as biological binding agents that stabilize soil particles and improve soil structure. This biological aggregation process not only enhances water retention and erosion resistance but also increases the persistence of carbon within the soil matrix [164].

Recent studies further indicate that ecosystem recovery depends on the re-establishment of belowground ecological networks rather than the recovery of individual microbial taxa alone. Analyses of restored ecosystems have revealed that increasing connectivity among bacteria, fungi, and other soil organisms is often associated with greater ecosystem multifunctionality and resilience. These observations support the concept that restoration should focus on rebuilding ecological interactions and functional networks capable of sustaining ecosystem processes under changing environmental conditions [165,166].

Carbon sequestration is also increasingly linked to regenerative agricultural practices. Long-term implementation of cover cropping, reduced tillage, organic amendments, and diversified cropping systems has been associated with greater microbial activity, enhanced carbon inputs, and increased soil organic carbon accumulation. Such practices not only improve soil health but also contribute to climate-change mitigation by promoting the transfer of atmospheric carbon into relatively stable soil reservoirs [167,168].

## 6. Challenges and Future Perspectives

### 6.1. Field Variability and Colonization Stability

One of the major challenges in soil microbiome engineering is achieving consistent performance under field conditions. While many microbial interventions show promising results in greenhouse or laboratory experiments, their effectiveness often declines when exposed to the environmental heterogeneity of agricultural systems [169]. This limitation arises because soil microbiomes are not passive recipients of introduced microorganisms. Rather, they represent historically assembled, spatially structured, and functionally complex communities shaped by local soil properties, plant hosts, resource availability, climatic conditions, and previous management practices [170]. Consequently, introduced microorganisms must compete with established resident communities and adapt to highly variable ecological conditions, which can strongly influence colonization success and long-term persistence.

The importance of ecological context was demonstrated in a transplantation study of squash root microbiomes originating from historically arid and humid environments. Even after transfer to a common-garden setting, microbial communities retained distinct taxonomic and functional characteristics. Arid-derived microbiomes remained enriched in genes associated with protein folding, oxidative stress tolerance, compatible solute synthesis, and osmotic regulation, indicating the persistence of environmental adaptation signatures. These findings suggest that microbial communities can preserve ecological memory following transplantation, but they also highlight the importance of matching introduced microbiomes with compatible environmental conditions [85,171].

Field reproducibility is further complicated by the fact that many agricultural interventions influence entire microbial and metabolic networks rather than individual taxa. A multi-omics investigation of carrots cultivated in soils amended with thermophile-fermented compost showed that improvements in productivity and crop quality were accompanied by coordinated shifts in soil bacterial communities and metabolite profiles. Structural equation modelling identified *Paenibacillus* and several amino-acid-related metabolites as important components of these responses [172]. Such results indicate that successful microbiome manipulation often depends on network-level ecological reorganization rather than the establishment of a single beneficial microorganism.

Similar complexity has been observed in large-scale soil restoration strategies. A four-year field trial evaluating enhanced weathering with crushed basalt demonstrated simultaneous improvements in carbon sequestration, soil fertility, and crop productivity [172]. In addition to increased maize and soybean yields, the treatment reduced soil acidification and stimulated the expression of root nutrient transport genes. These findings illustrate that biological responses in agricultural soils are frequently shaped by interactions among microbial communities, mineral amendments, plant physiology, and environmental conditions, making long-term outcomes difficult to predict from individual factors alone.

Long-term climate experiments provide additional evidence that microbial communities are inherently dynamic. By incorporating microbial functional gene abundance into a decomposition model, one study demonstrated that microbial thermal adaptation reduced respiration-driven carbon loss under warming conditions and significantly improved predictions of soil carbon dynamics. These findings indicate that microbial communities continuously adjust to environmental change, meaning that an intervention considered successful at one stage may perform differently as microbial adaptation proceeds over time [173].

#### Why Microbial Inoculants Fail Under Field Conditions

Despite promising results under laboratory and greenhouse conditions, microbial inoculants frequently show weak, inconsistent, or non-significant effects in field experiments. For example, a recent maize field experiment showed that fertilization regime exerted a stronger influence on the indigenous rhizosphere microbiome than the applied microbial consortia, while consortium application did not significantly alter plant-growth-promoting functional profiles. This finding illustrates how management context and resident-community resistance can override the expected effects of microbial inoculation. This failure does not necessarily indicate that the selected microorganisms lack beneficial traits. Rather, introduced strains must establish within a resident microbiome that is already adapted to the local soil, crop, climate, and management history. As a result, successful performance in simplified experimental systems often does not translate directly to heterogeneous agricultural soils [72,169].

Poor establishment is one of the most common causes of field failure. Introduced microorganisms may be unable to occupy suitable ecological niches, compete effectively for root-colonization sites, or maintain sufficient population density to express the intended function. Even multi-strain inoculants and SynComs may lose members after application, resulting in changes in community composition, loss of functional complementarity, and reduced performance. Soil pH, moisture, temperature, nutrient availability, crop genotype, and application timing further influence inoculant survival and activity [31,71,72].

Biotic interactions can also strongly influence inoculant persistence. Bacteriophages may infect and suppress introduced bacterial populations, whereas predatory protists can selectively consume bacterial cells and alter the structure of the applied community. These top-down controls are rarely represented in sterile soils or simplified greenhouse systems but may substantially alter inoculant abundance and functional stability under field conditions [49,115].

Competition for limiting resources represents another important constraint. Iron is particularly relevant because both beneficial microorganisms and resident competitors rely on siderophores and other iron-acquisition systems. An introduced strain may possess plant-growth-promoting traits but still fail if indigenous microorganisms capture iron more efficiently [73]. In addition, microbial degradation or exploitation of plant-derived iron-mobilizing compounds may enhance bacterial persistence while reducing iron availability to the plant, illustrating that successful colonization does not always result in a beneficial plant response [58].

Field performance may also be limited by formulation and delivery. Declining viability during storage, desiccation after application, incompatibility with fertilizers or pesticides, insufficient carrier protection, and poor synchronization with root development can reduce the number of active cells reaching the target niche. Therefore, failure may occur before ecological interaction with the resident microbiome is fully established [70,72].

These limitations indicate that inoculant performance should not be evaluated only through short-term plant-growth responses. Field trials should monitor strain persistence, changes in community composition, functional gene expression, and agronomic outcomes across multiple soils, crop genotypes, locations, and growing seasons. Unsuccessful or neutral field trials should also be reported because they provide essential information for identifying ecological barriers and improving future inoculant design [14,169,170].

### 6.2. Biosafety, Regulation, and Commercialization

The successful implementation of soil microbiome technologies depends not only on scientific effectiveness but also on biosafety, regulatory approval, and commercial feasibility. While microbial inoculants, SynComs, and engineered microorganisms offer considerable potential for sustainable agriculture, their introduction into open environments raises concerns regarding ecological impacts, persistence, horizontal gene transfer, and unintended effects on native microbial communities [174,175].

Regulatory frameworks currently differ among countries and are often better developed for conventional biofertilizers than for engineered microorganisms or microbiome-based products. As a result, commercialization can be slowed by uncertainty regarding risk assessment, registration procedures, and environmental monitoring requirements [176].

Artificial microbial communities require particular regulatory attention because their properties cannot always be predicted from the characteristics of individual strains alone. Regulatory assessment should therefore include accurate identification of all community members, confirmation of community composition and stability, evaluation of strain–strain interactions, and demonstration of consistent biological activity. For genetically modified microorganisms, additional requirements should include molecular characterization of the introduced genetic elements, assessment of genetic stability, verification of biological containment measures, and authorization before deliberate environmental release [174,175,176].

Environmental risk assessment should evaluate the persistence and dispersal of introduced microorganisms, their potential for horizontal gene transfer, and possible effects on non-target organisms and indigenous soil microbial communities. Particular attention should also be given to unintended changes in nutrient cycling, disease-suppressive functions, and other ecosystem processes. A stepwise assessment approach involving laboratory studies, greenhouse experiments, confined field trials, and post-release monitoring would help identify potential risks before large-scale agricultural application [174,175].

Another challenge is product consistency. Microbial strains that perform well under laboratory conditions may exhibit reduced survival, colonization, or functionality under field conditions, leading to variable commercial performance. Therefore, successful commercialization requires not only effective microbial formulations but also robust production systems, quality control standards, and long-term field validation [177].

Future progress will depend on the development of harmonized regulatory guidelines, improved biosafety assessment frameworks, post-release monitoring procedures, and scalable manufacturing technologies.

### 6.3. Data Integration and Translational Limitations

The rapid adoption of high-throughput sequencing, metabolomics, metatranscriptomics, proteomics, and environmental sensing technologies has generated unprecedented amounts of soil microbiome data. However, converting these datasets into actionable agricultural solutions remains a major challenge. One of the primary limitations is that different omics platforms capture distinct layers of biological organization, making integration difficult and often resulting in inconsistent interpretations across studies [178,179].

Recent investigations have demonstrated that microbial taxonomic composition alone is frequently insufficient to predict ecosystem functions. Multi-omics studies have revealed that similar microbial communities can exhibit substantially different metabolic activities depending on environmental conditions, host genotype, or management practices. Consequently, linking microbial diversity directly to agronomic outcomes remains challenging because microbial functions are context-dependent and dynamically regulated [180].

Another important limitation concerns data standardization. Differences in sampling strategies, DNA extraction methods, sequencing platforms, bioinformatic pipelines, and metadata collection can substantially affect study outcomes. Large comparative analyses have shown that methodological variation often contributes significantly to differences observed among microbiome datasets, limiting reproducibility and cross-study comparisons [181].

The growing application of ML and AI has improved predictive modelling of soil microbiomes, yet translational barriers remain. Many predictive models perform well within specific datasets but exhibit reduced accuracy when applied across locations, crop species, or climatic regions. This limitation arises because most models are trained on geographically restricted datasets and frequently fail to capture the ecological complexity of real agricultural systems [182].

Furthermore, the gap between correlation and causation remains a persistent challenge. While large-scale microbiome surveys can identify microbial taxa associated with desirable traits, these associations do not necessarily indicate causal relationships. Recent studies increasingly emphasize the need for synthetic community experiments, microbiome transplantation, and functional validation to confirm the biological relevance of candidate microorganisms identified through computational analyses [183].

Therefore, future progress will depend on the development of standardized workflows, interoperable databases, integrated multi-omics frameworks, and experimentally validated predictive models. Bridging the gap between data generation and practical agricultural application remains one of the most important challenges facing soil microbiome research.

### 6.4. Future Directions in Precision Microbiome Engineering

The future of soil microbiome engineering is expected to move beyond the introduction of individual microbial strains toward the precise manipulation of entire microbial networks and ecosystem functions. Advances in multi-omics technologies, AI, synthetic biology, and precision agriculture are increasingly enabling the design of microbiome interventions tailored to specific crops, soils, and environmental conditions [184,185].

One emerging direction is the development of function-oriented SynComs. Rather than selecting microorganisms based solely on taxonomic identity, recent studies emphasize assembling consortia according to complementary ecological functions such as nitrogen fixation, phosphorus mobilization, pathogen suppression, drought tolerance, and carbon stabilization. This approach aims to improve colonization stability and functional reliability under field conditions [186].

Precision microbiome engineering is also becoming increasingly integrated with real-time environmental monitoring. Advances in soil sensors, remote sensing technologies, and machine learning algorithms now allow continuous assessment of soil moisture, nutrient availability, salinity, and biological activity. Such information may eventually support adaptive microbiome management strategies in which microbial interventions are deployed according to site-specific environmental conditions [187].

Another promising area involves host-directed microbiome engineering. Recent evidence indicates that plant genotypes differ substantially in their ability to recruit beneficial microorganisms. Consequently, future breeding programs may incorporate microbiome-associated traits alongside traditional agronomic characteristics, enabling crops to more effectively establish beneficial microbial partnerships under stress conditions [35,188].

Synthetic biology- and CRISPR-based technologies are expected to further expand the field by enabling the development of programmable microbial systems with improved nutrient-use efficiency, environmental responsiveness, and biosafety features. Although large-scale agricultural deployment remains limited, recent advances in microbial chassis engineering and genetic containment systems suggest that engineered microorganisms may eventually complement naturally occurring microbiomes in specific applications [189].

At a broader scale, future microbiome engineering strategies are likely to incorporate ecological principles such as community assembly, microbial succession, trophic interactions, and ecosystem resilience. Rather than attempting to control individual microorganisms, emerging frameworks seek to guide the self-organization of microbial communities toward desirable functional states [190].

Ultimately, the next generation of precision microbiome engineering will depend on integrating ecological theory, multi-omics data, predictive modelling, and field-scale validation. Such approaches have the potential to transform soil microbiome management from empirical experimentation into a predictive and adaptive technology capable of supporting sustainable agriculture, climate resilience, and long-term ecosystem health.

### 6.5. Cross-Cutting Mechanisms, Controversies, and Unresolved Questions

Across the available evidence, several recurring ecological principles appear to determine the outcome of soil microbiome interventions. Host-mediated selection is a major driver of microbiome assembly because plant genotype, root architecture, and other heritable traits influence the recruitment of microorganisms from the surrounding soil [173,175]. Resource and metabolite exchange further regulates microbial establishment, as root-derived compounds can either recruit beneficial microorganisms or be exploited by microbes in ways that do not benefit the host plant [57,58]. Community-level functions also depend on interactions among microorganisms, including cross-feeding, competition, trophic regulation, and context-dependent changes in microbial phenotypes [112,114]. In parallel, soil physicochemical conditions act as strong environmental filters, as nutrient availability, pH, and climatic history can determine whether beneficial microbial functions are expressed [46,69].

These mechanisms explain why the same microbial taxon may produce different outcomes in different plant or soil environments. For example, the functional role of an individual microorganism can change depending on the composition of the surrounding community, indicating that microbial traits cannot always be predicted from isolated strains alone [114]. Similarly, resilient plant-associated communities may include both a stable core microbiota and stress-responsive taxa that perform different but complementary ecological roles [68]. Thus, successful microbiome engineering may depend less on maintaining an identical taxonomic composition than on preserving key functions and interactions within the community [71,112].

A major unresolved question is whether stable colonization by introduced microorganisms is required for long-term agronomic benefits. Many inoculants perform successfully under laboratory or greenhouse conditions but show reduced persistence and inconsistent effects under heterogeneous field conditions [72,169]. However, a microorganism may temporarily modify plant physiology or the resident community before declining in abundance. Therefore, taxonomic persistence and functional persistence should be evaluated as distinct outcomes rather than treated as equivalent indicators of intervention success [18,72,169].

Increasing microbial diversity also does not necessarily result in greater functional performance. Experiments with reduced-complexity communities have shown that initial richness affects community succession and stabilization, but the outcome also depends strongly on the physical environment in which the community develops [113]. Highly complex communities may provide functional redundancy and resilience, but they may also contain competitors or antagonists that reduce the intended function [105,112]. In contrast, simplified SynComs are easier to characterize and experimentally control, although their stability under variable field conditions remains uncertain [71].

Another unresolved issue concerns the relative effectiveness of direct and indirect microbiome management. Direct interventions, including microbial inoculation, SynCom application, microbiome transplantation, and engineered microorganisms, provide greater control over the biological material being introduced [14,91,169]. Nevertheless, these approaches frequently encounter poor establishment and competition with indigenous microbial communities under field conditions [18,72]. Indirect approaches, such as selecting plant genotypes with favorable microbiome-recruitment traits or modifying soil management practices, may promote microorganisms already adapted to local conditions [36,47,173]. However, these effects are less predictable and may be difficult to separate from the direct effects of plant traits or soil management [45,47].

Resolving these controversies will require comparative experiments across multiple soils, crop genotypes, climatic regions, and growing seasons [169,170]. Standardized sampling and analytical workflows are needed because methodological differences currently limit reproducibility and cross-study comparison [166]. In addition, associations identified through microbiome profiling or predictive modelling should be validated using microbial isolation, SynCom reconstruction, transplantation, or controlled perturbation experiments [113,114,168]. Such studies should measure both microbial persistence and functional outcomes, including crop productivity, stress resilience, and soil ecosystem processes under realistic field conditions [18,169,170].

## 7. Global Research Trends and Knowledge Structure of Soil Microbiome Engineering

To identify the major research trends and thematic structure of soil microbiome engineering, a bibliometric keyword co-occurrence analysis was conducted using VOSviewer (version 1.6.20). Bibliographic records were retrieved from the Scopus database on 30 June 2026. The search was restricted to English-language research articles published between 2020 and 2026. The following search query was used:

TITLE-ABS-KEY (“soil microbiome engineering” OR “soil microbiome” OR “rhizosphere microbiome” OR “microbiome engineering” OR “rhizosphere engineering”) AND PUBYEAR >2019 AND LIMIT-TO (DOCTYPE, “ar”) AND LIMIT-TO (LANGUAGE, “English”)

A total of 293 documents were retrieved and exported in CSV format, including bibliographic information, author keywords, indexed keywords, citation data, and abstracts. The dataset was imported into VOSviewer, and keyword co-occurrence analysis was performed using all keywords as the unit of analysis and the full counting method. Generic indexing terms such as “article”, “nonhuman”, and “controlled study” were excluded during manual data cleaning to improve thematic interpretation. A minimum keyword occurrence threshold of two was applied, resulting in the selection of 18 highly connected keywords for network visualization and cluster analysis.

The keyword frequency and total link strength (TLS) values are summarized in Table 3. The most frequently occurring terms were Microbiology, Microbiota, Microflora, and Soil microbiology, each appearing six times and exhibiting the highest TLS (95), indicating their central role within the research network (Table 3). Rhizosphere was also highly represented, with six occurrences and a TLS value of 81, reflecting the importance of root-associated microbial communities in soil microbiome engineering studies (Table 3). Other influential terms included Microbiome (5 occurrences, TLS = 61), Microbial community (4 occurrences, TLS = 75), and Rhizosphere microbiome (4 occurrences, TLS = 46), highlighting the increasing focus on microbial community structure and plant-associated microbiomes (Table 6).

Several functionally relevant terms, including Nitrogen, Biomass, Enzyme activity, *Actinobacteria*, and *Bacillus*, were also identified among the most connected keywords, suggesting a strong emphasis on nutrient cycling, microbial functionality, and beneficial microorganisms in current research. Furthermore, the occurrence of keywords such as Plant–microbe interactions and Plant growth indicates growing interest in applying microbiome engineering strategies to improve crop productivity and sustainable agriculture.

**Figure 4:**
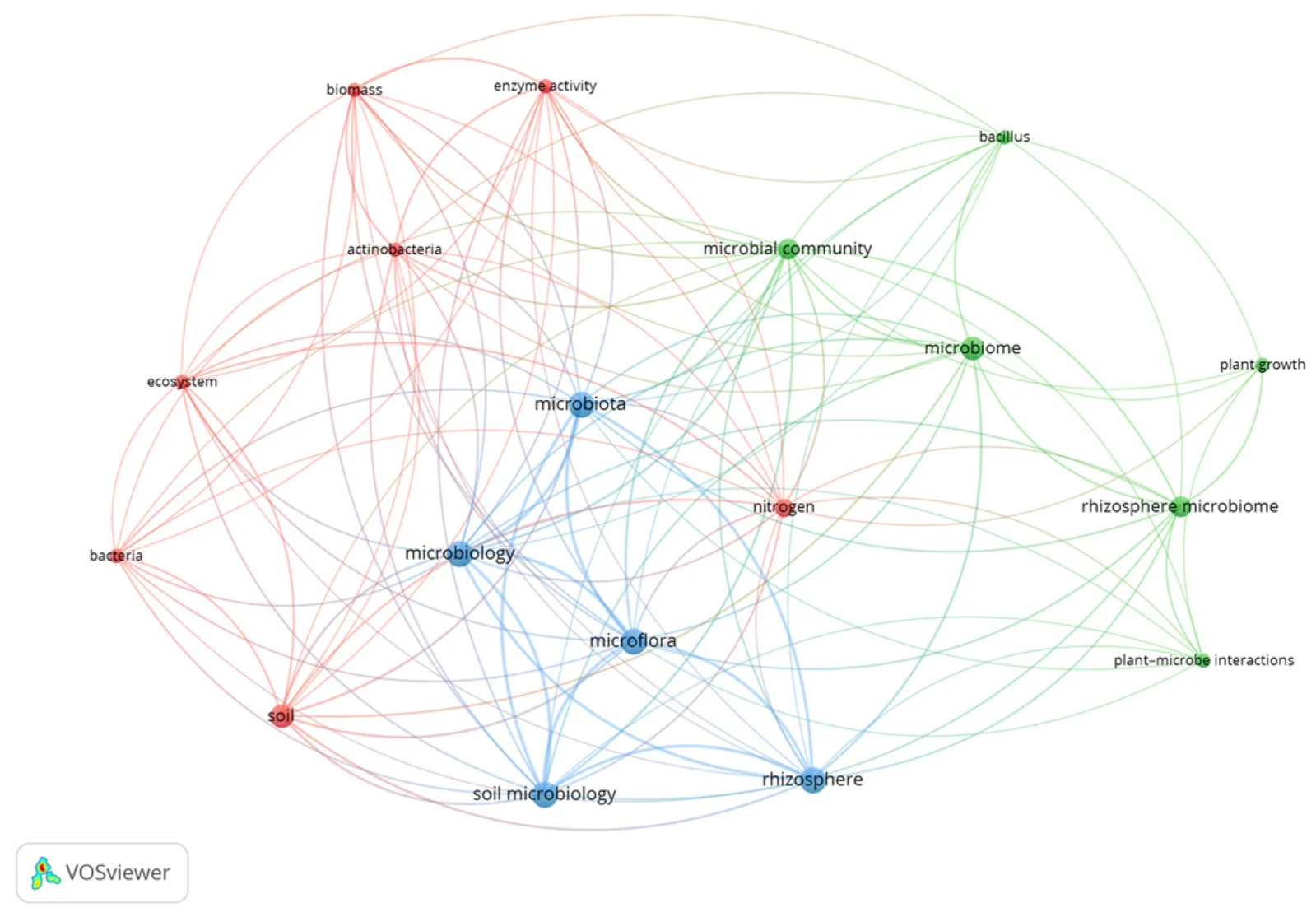
Keyword co-occurrence network generated using VOSviewer based on the selected literature on soil microbiome engineering. Node size reflects keyword frequency, line thickness indicates co-occurrence strength, and colors represent thematic clusters.

The network visualization revealed three major thematic clusters within the soil microbiome engineering literature (Figure 4). The first cluster (blue) was centered around Microbiology, Microbiota, Microflora, Soil microbiology, and Rhizosphere, representing studies focused on microbial diversity, community composition, and rhizosphere ecology. The second cluster (green) included Microbiome, Microbial community, Rhizosphere microbiome, *Bacillus*, Plant growth, and Plant–microbe interactions, reflecting research aimed at microbiome manipulation and plant growth promotion. The third cluster (red) comprised Soil, Nitrogen, Biomass, Enzyme activity, Actinobacteria, Bacteria, and Ecosystem, representing investigations into soil ecosystem functioning, nutrient cycling, and microbial contributions to ecosystem services. Together, these clusters demonstrate that contemporary soil microbiome engineering research integrates microbial ecology, plant health, and ecosystem functionality within a multidisciplinary framework.

**Figure 5:**
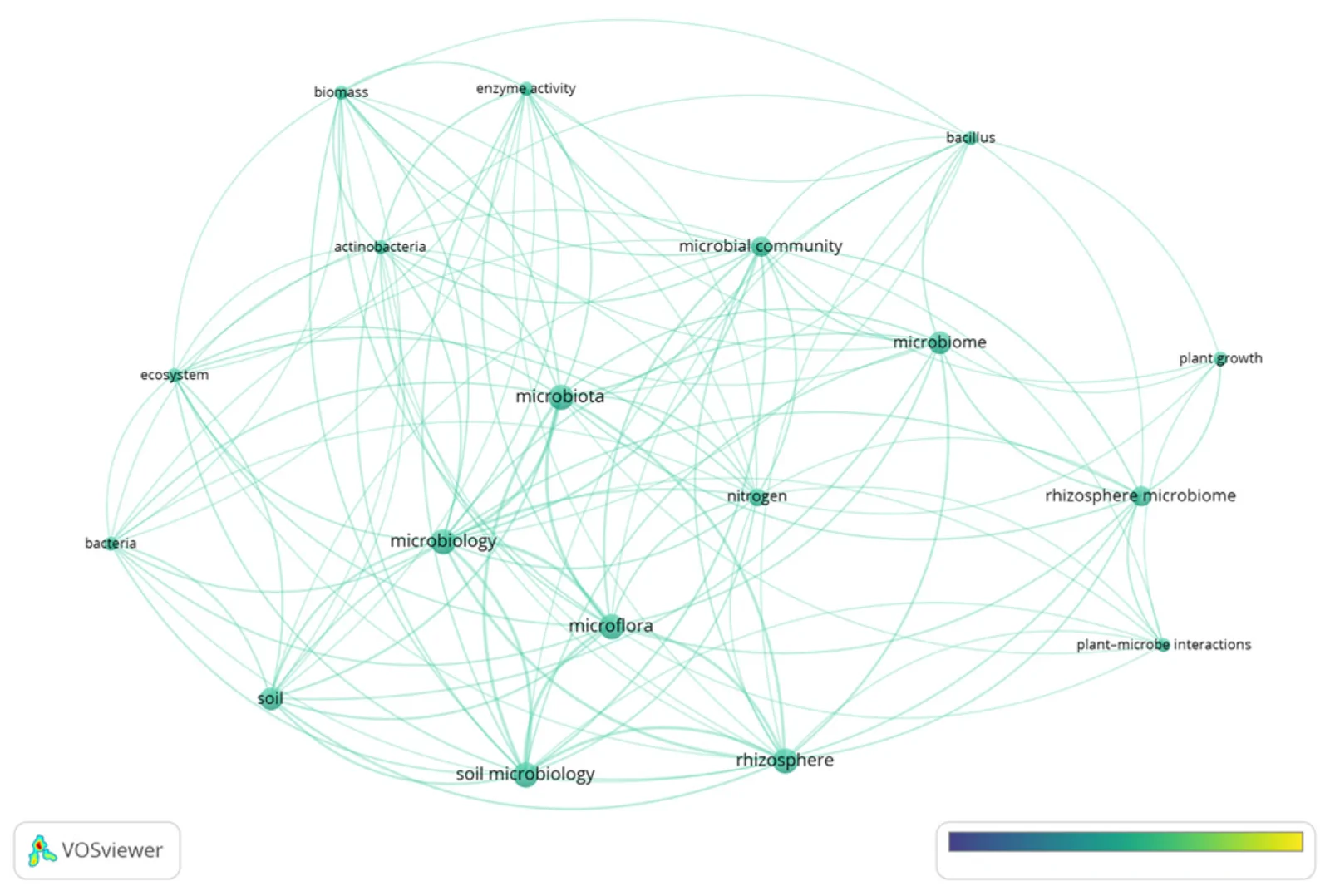
Overlay visualization of keyword relationships in soil microbiome engineering research generated using VOSviewer. The map illustrates the overall connectivity among the selected keywords.

The overlay visualization further demonstrated the interconnected nature of the identified keywords within the research field (Figure 5). Central terms such as Microbiota, Microbiology, Microflora, Rhizosphere, and Microbiome occupied dominant positions in the network, indicating that these concepts serve as the core knowledge base underpinning soil microbiome engineering research. The strong connectivity among these terms suggests a high degree of integration between classical soil microbiology and emerging microbiome-focused approaches.

**Figure 6:**
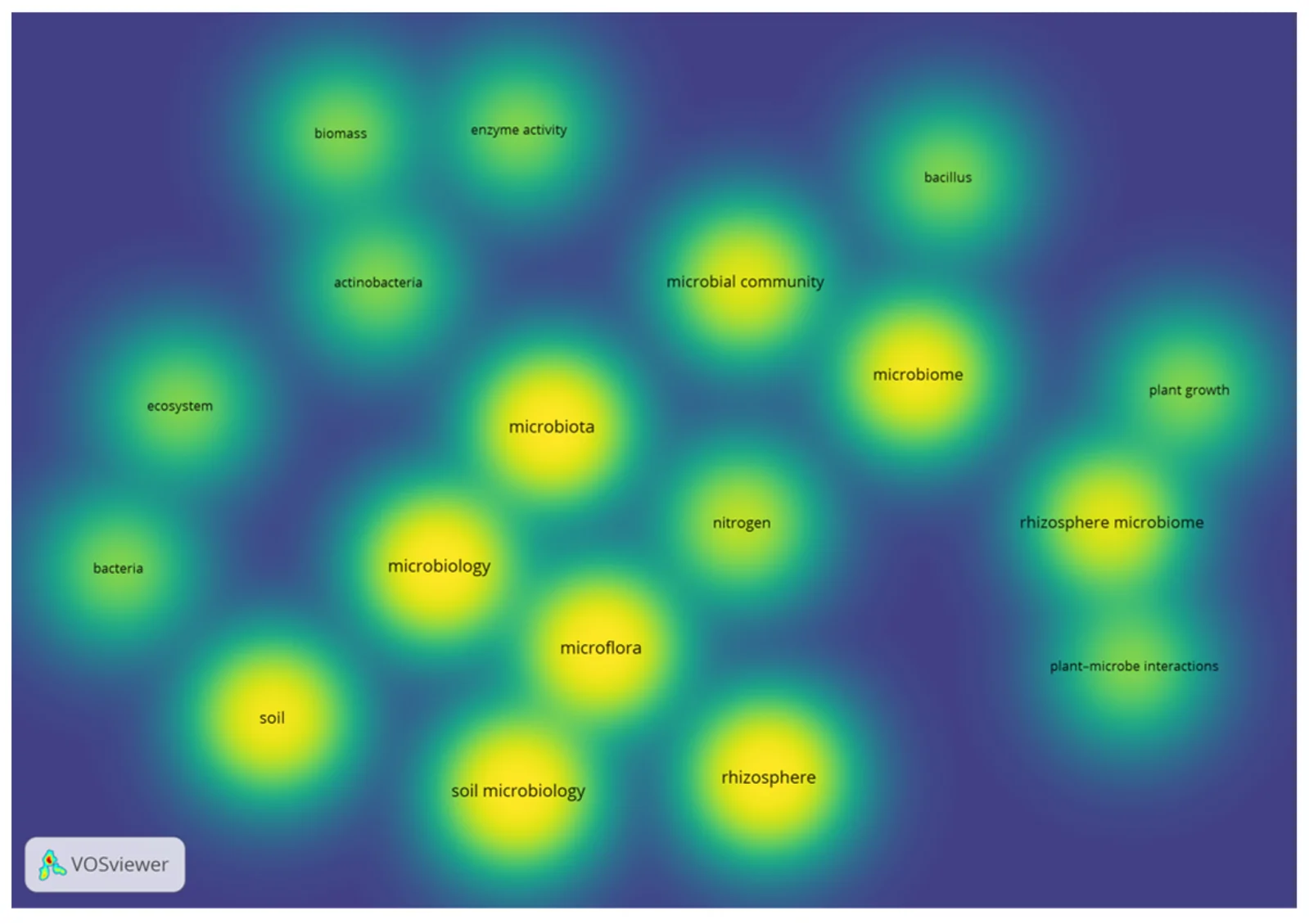
Density visualization of keyword co-occurrence in soil microbiome engineering research generated using VOSviewer. Yellow regions indicate the highest concentration of interconnected research topics, whereas green and blue regions represent areas of lower thematic density.

The density visualization highlighted the principal research hotspots within the soil microbiome engineering field (Figure 6). The highest-density regions were centered around Microbiology, Microbiota, Microflora, Soil microbiology, Rhizosphere, and Microbiome, indicating that these topics constitute the major knowledge hubs of the discipline. Moderate-density regions included Microbial community, Rhizosphere microbiome, Nitrogen, and *Bacillus*, reflecting their growing importance in contemporary research. In contrast, plant growth, Plant–microbe interactions, Ecosystem, and Bacteria appeared in lower-density regions, suggesting more specialized but emerging research directions. Overall, the density map confirms that rhizosphere-associated microbiomes, microbial ecology, and soil microbial communities remain the dominant focus areas in soil microbiome engineering research.

The VOSviewer analysis indicates that soil microbiome engineering has evolved from descriptive studies of soil microbial diversity toward more application-oriented research emphasizing rhizosphere microbiomes, microbial community engineering, nutrient cycling, and plant growth promotion. These trends highlight the transition of the field toward precision microbiome management strategies for sustainable agricultural production and ecosystem resilience.

## 8. Conclusions

The soil microbiome plays a central role in sustainable agriculture by regulating nutrient cycling, plant growth, disease suppression, stress tolerance, and ecosystem functioning. Advances in microbial ecology have shifted the perception of soil microorganisms from passive inhabitants to active drivers of plant health and agroecosystem resilience. As discussed throughout this review, soil microbiome assembly is shaped by complex interactions among plant genetics, environmental conditions, agricultural practices, and microbial ecological processes. Recent developments in microbiome engineering have expanded available approaches beyond traditional microbial inoculants. Strategies such as SynComs, microbiome transplantation, rhizosphere engineering, and synthetic biology offer new opportunities to manipulate microbial functions in a more targeted and predictable manner. In parallel, the integration of metagenomics, metabolomics, metatranscriptomics, systems biology, and AI has substantially improved our ability to characterize microbial communities and predict their responses under diverse environmental conditions. The bibliometric analysis revealed that current research is strongly focused on microbiology, microbiota, microflora, rhizosphere ecology, and microbiome-related processes, reflecting the transition from descriptive microbial ecology toward application-oriented microbiome management. Growing interest in microbial community engineering, nutrient cycling, plant growth promotion, and climate adaptation highlights the increasing importance of microbiome-based technologies for sustainable crop production. This review uniquely integrates current knowledge on soil microbiome assembly, ecological functions, engineering approaches, multi-omics technologies, AI, and sustainable agricultural applications into a single conceptual framework. In addition, the bibliometric analysis provides quantitative evidence of evolving research priorities, emerging themes, and existing knowledge gaps in soil microbiome engineering. Such a holistic perspective helps bridge the gap between fundamental microbial ecology and the practical implementation of microbiome-based solutions for climate-smart and sustainable crop production. Despite significant progress, several challenges continue to limit large-scale implementation, including environmental heterogeneity, instability of introduced microorganisms, competition with native microbial communities, biosafety concerns, and difficulties in integrating complex multi-omics datasets. Addressing these limitations will require long-term field validation, standardized methodologies, and improved predictive frameworks. Future soil microbiome engineering is expected to move toward precision microbiome management that combines ecological principles, host genetics, real-time monitoring, AI, and synthetic biology. Such advances may enable the development of resilient, climate-smart agricultural systems with reduced dependence on chemical inputs and improved ecosystem sustainability.

## Figures and Tables

**Table 1:** Mechanistic insights into soil microbiome assembly across the soil–plant continuum.

Biological System	Experimental Design and Methods	Main Findings on Structure and Diversity	Contribution to the Topic	Ref.
*Medicago truncatula*; 3 genotypes; 2 native soils	Common-garden experiment; 16S rRNA amplicons + shotgun metagenomics	Diversity decreased from rhizosphere to internal compartments; soil origin dominated rhizosphere, genotype dominated root endosphere	Established a compartment-specific filtering framework for plant-associated soil microbiomes	[35]
Field-grown wheat cultivars from tall to semi-dwarf	Field phenotyping + rhizosphere bacterial profiling	Breeding-related root trait shifts were associated with distinct bacterial assemblages	Showed that crop improvement history leaves a structural signature on the rhizosphere microbiome	[36]
39 tetraploid wheat accessions across domestication gradient	Rhizospheric bacterial/fungal profiling + N-cycling guild quantification	Domestication groups differed in rhizosphere community structure and nitrogen-related guild abundance	Linked domestication history to microbiome configuration and N-relevant ecology	[37]
Maize at two sites and three developmental stages	432 samples; bacterial and fungal amplicon sequencing + metagenomics	Developmental stage was a stronger driver in plant compartments than in soils; temporal shifts changed interkingdom networks and functions	Demonstrated that microbiome diversity is temporally dynamic and developmentally staged	[38]
Wheat rhizosphere assembled from soil and seed-borne bacteria	Sequential propagation in sterilized microcosms; functional analysis of resulting community	Stable rhizobiome emerged from soil–seed coalescence; seed-borne taxa contributed facilitative traits	Added vertical microbial inheritance to modern rhizobiome assembly models	[39]

**Table 2:** Representative mechanistic studies on soil microbiome functions in agroecosystems.

Agroecosystem Function Targeted	Experimental Design and Methods	Principal Mechanistic Findings	Agronomic Significance	Ref.
Sustained productivity under nutrient imbalance	Long-term fertilization field trial; quantitative microbiome profiling; metagenomics; SynCom validation	Nitrogen omission enriched a specific bacterial cluster; SynCom derived from that cluster promoted soybean growth	Showed that root microbiome succession can buffer nutrient deficiency and sustain yield	[43]
Carbon cycling in maize rhizosphere	Long-term fertilization experiment; rhizosphere metagenomics	rTCA was dominant C-fixation pathway; N fertilization stimulated methanogenesis-related functions; straw return enhanced C degradation genes	Linked fertilization strategy to microbial control of C fixation, decomposition, and CH_4_ metabolism	[44]
Depth-stratified microbial assembly under management	16S/ITS sequencing from 0–60 cm; ML; co-occurrence and neutral models	Fertility source was strongest driver, especially for fungi; tillage increased shallow-soil stochasticity	Demonstrated that microbiome function and assembly must be interpreted across soil depth, not only topsoil	[45]
Natural disease suppression	Field survey + microbiome transfer assays + metagenomics + metabolomics	Soil acidification reduced anti-*Fusarium* capacity and downregulated sulfur-metabolism-related functions	Established pH as a determinant of whether a soil microbiome can remain suppressive	[46]
Plant defense and pest suppression	136 organic farm soils; pea bioassays; ML; structural equation modeling	No tillage, targeted irrigation, and grass cover crops were linked to microbiome states associated with higher JA/SA and lower aphid pressure	Reconnected belowground microbiome management with aboveground defense outcomes	[47]
Nutrient cycling and yield in ginger	16S/ITS amplicons + LC-MS/MS metabolomics across contrasting cultivars	High-yield cultivar had more complex networks and metabolite-linked keystone taxa (*Talaromyces*, *Devosia*)	Showed that productivity depends on coordinated host-metabolite–microbiome organization	[48]
Suppressive soil through food-web activation	Field biofertilizer experiment; focus on protists and secondary-metabolite genes	Predatory protists, especially *Cercomonas*, increased with biofertilizer; PKS-related functions rose as disease incidence fell	Demonstrated that successful suppressiveness can involve trophic regulation, not just bacterial antagonism	[49]

Note: ITS, Internal Transcribed Spacer; LC-MS/MS, Liquid Chromatography–Tandem Mass Spectrometry; ML, Machine Learning; rTCA, Reductive Tricarboxylic Acid Cycle; SynCom, Synthetic Microbial Community; JA, jasmonic acid; SA, salicylic acid; PKS, polyketide synthase.

**Table 3:** Principal functions of beneficial bacteria in soil microbiome engineering.

Functional Category	Representative Bacterial Genera	Principal Mechanisms	Relevance to Microbiome Engineering	Ref.
Nutrient acquisition	*Rhizobium, Azospirillum, Paenibacillus*	Nitrogen fixation, phosphate solubilization, siderophore-mediated Fe acquisition	Reduction in mineral fertilizer requirements and improvement of nutrient-use efficiency	[85]
Plant growth and stress mitigation	*Bacillus, Pseudomonas, Paenibacillus*	Phytohormone production, ACC deaminase activity, osmotic adjustment and root development	Improved tolerance to salinity, drought and nutrient limitation	[77,86]
Disease suppression	*Bacillus, Pseudomonas, Streptomyces, Lysobacter*	Antibiotics, lytic enzymes, nutrient competition, volatile compounds and induced systemic resistance	Biological control of soil-borne pathogens and activation of host immunity	[87]
Root colonization and persistence	*Bacillus, Pseudomonas, Rhizobium*	Chemotaxis, quorum sensing, biofilm formation and utilization of root-derived metabolites	Improved establishment and interaction with the resident microbiome	[88]
Synthetic bacterial communities	Functionally complementary bacterial strains	Cross-feeding, functional redundancy, division of labor and emergent metabolite production	More controllable and potentially more stable functions than single-strain inoculants	[89]

Note: ACC, 1-aminocyclopropane-1-carboxylic acid; Fe, iron.

**Table 4:** Critical comparison of soil microbiome intervention strategies.

Strategy	Defining Feature	Evidence and Examples Represented in This Review	Principal Advantage	Principal Limitation or Uncertainty	Ref.
Conventional microbial inoculants	Introduction of selected bacterial or fungal strains, or relatively simple microbial preparations, to provide a specific plant-growth-promoting, biocontrol, or stress-protective function	Microbial inoculants can modify resident soil communities. Combined application of indigenous arbuscular mycorrhizal fungi and compost improved drought tolerance in carob, whereas *Lysobacter capsici* AZ78 produced antimicrobial metabolites with biocontrol potential	Relatively straightforward formulation and the possibility of targeting a defined plant-growth-promoting, biocontrol, or stress-protective function	Establishment and performance depend strongly on soil conditions, host species, native microbiota, formulation, and ecological competition; beneficial effects observed under controlled conditions may not be reproduced consistently in the field	[72,78,79]
Function-oriented SynComs	Defined microbial assemblages intentionally constructed according to complementary functions and ecological interactions	A soybean-derived SynCom reproduced growth-promoting effects under nitrogen omission, whereas a four-member Gram-positive bacterial SynCom protected pepper plants through community-dependent volatile production. These studies illustrate how function-oriented design can generate complementary and emergent microbial activities	Greater compositional control than natural communities, functional complementarity, and suitability for causal and mechanistic experiments	Strain compatibility, community succession, initial abundance ratios, and environmental context can alter the expected function; stability across soils and growing seasons remains uncertain	[43,71,83]
Rhizobiome transplantation	Transfer of a complex or relatively intact donor rhizosphere community rather than individual strains	Protective rhizobiome transplantation has been proposed as an alternative to single-strain inoculation. Squash microbiomes originating from arid environments retained taxonomic and functional traits associated with osmotic adaptation after transplantation	Preserves microbial interactions, functional diversity, and potentially the ecological memory of the donor microbiome	Low control over exact community composition, strong dependence on donor and recipient soils, and difficulties in standardization, reproducibility, storage, and regulatory characterization	[90,91,92]
Indirect rhizosphere steering and host-mediated manipulation	Modification of plant traits, root-associated conditions, litter chemistry, mycorrhizal associations, or soil resources to favor beneficial native microbial communities	Host genotype and root traits influence microbial recruitment, whereas plant mycorrhizal association can alter soil microbial biomass and metabolic activity. Fungal hyphal networks may further support microbial dispersal and metabolite movement within the rhizosphere	Uses resident microbial communities and may therefore provide greater ecological compatibility than the introduction of non-native strains	Effects are indirect, context-dependent, and often slower to develop; microbiome-mediated effects may be difficult to distinguish from direct plant or soil responses	[36,93,95]
Engineered microorganisms and synthetic biology-based interventions	Genetic modification of microbial strains or communities using CRISPR, synthetic biology, metabolic engineering, or programmable regulatory systems	Regulatory control of nitrogen fixation has been investigated in cereal-associated bacteria, and CRISPR/Cas9 tools have been developed for rapid and iterative genome editing in *Pseudomonas putida* KT2440	The highest level of functional precision and the possibility of programming nutrient exchange, environmental responsiveness, and specific metabolic pathways	Host-dependent gene expression, metabolic burden, genetic instability, competition with native microorganisms, containment, biosafety, and regulatory uncertainty; most applications remain at laboratory or contained-experiment stages	[99,100,101]

Note: SynCom, synthetic microbial community; CRISPR, clustered regularly interspaced short palindromic repeats.

**Table 5:** Representative AI and machine learning applications in soil microbiome research.

Prediction Objective	Input Data	Model or Analytical Approach	Main Practical Output	Main Limitation	Ref.
Prediction of potato disease and yield	Bacterial and fungal microbiome profiles combined with soil physicochemical variables	Random forest and Bayesian neural network	Pre-planting prediction of disease risk and crop performance	Microbiome data were more informative for some disease outcomes than for yield; performance depended strongly on preprocessing, environmental variables, and label definition	[120]
Identification of drought-responsive microbial indicators	Soil microbial abundance and environmental data	Interpretable ML with SHAP feature attribution	Identification and ranking of microbial taxa associated with drought response	Predictive importance indicates association but does not independently establish microbial function or causality	[121]
Large-scale assessment of crop health	Fungal soil microbiome profiles and multispectral satellite-derived vegetation indices	Two-stage ML framework	Connection of belowground fungal community patterns with remotely monitored crop-health indicators	Transferability across regions, crops, seasons, and remote-sensing platforms requires further validation	[123]
Prediction of bacterial and fungal community composition	Soil and environmental variables	Random forest, k-nearest neighbors, and multilayer perceptron	Prediction of microbiome composition from measurable environmental variables	Traditional algorithms may outperform deep learning when training datasets are small	[125]

Note: SHAP, Shapley Additive Explanations; ML, machine learning; AI, artificial intelligence.

**Table 6:** Most frequent keywords identified by VOSviewer co-occurrence analysis.

№	Keyword	Occurrences	TLS
1	Microbiology	6	95
2	Microbiota	6	95
3	Microflora	6	95
4	Soil microbiology	6	95
5	Rhizosphere	6	81
6	Microbial community	4	75
7	Microbiome	5	61
8	Soil	5	48
9	Rhizosphere microbiome	4	46
10	Actinobacteria	2	43
11	Biomass	2	43
12	Enzyme activity	2	43
13	Nitrogen	3	38
14	*Bacillus*	2	30
15	Bacteria	2	30
16	Ecosystem	2	29
17	Plant–microbe interactions	2	17
18	Plant growth	2	15

## Data Availability

No new data were created or analyzed in this study. Data sharing is not applicable to this article.

## Abbreviations

ACC	1-Aminocyclopropane-1-carboxylate
AI	Artificial Intelligence
AMF	Arbuscular Mycorrhizal Fungi
AMG	Auxiliary Metabolic Gene
CRISPR	Clustered Regularly Interspaced Short Palindromic Repeats
ITS	Internal Transcribed Spacer
JA	Jasmonic Acid
LC–MS/MS	Liquid Chromatography–Tandem Mass Spectrometry
ML	Machine Learning
OTU	Operational Taxonomic Unit
PGPB	Plant-Growth-Promoting Bacteria
PGPR	Plant-Growth-Promoting Rhizobacteria
PKS	Polyketide Synthase
qPCR	Quantitative Polymerase Chain Reaction
ROS	Reactive Oxygen Species
rTCA	Reductive Tricarboxylic Acid Cycle
SA	Salicylic Acid
SHAP	Shapley Additive Explanations
SynCom	Synthetic Microbial Community
